# Hemodialysis patient characteristics associated with better experience as measured by the In-center Hemodialysis Consumer Assessment of Healthcare Providers and Systems (ICH CAHPS) survey

**DOI:** 10.1186/s12882-018-1147-3

**Published:** 2018-11-28

**Authors:** Taimur Dad, Hocine Tighiouart, Eduardo Lacson, Klemens B. Meyer, Daniel E. Weiner, Michelle M. Richardson

**Affiliations:** 1 Tufts Medical Center, 800 Washington Street Box 391, Boston, MA 02111 USA; 2 Sackler School of Graduate Biomedical Sciences, Tufts University, Boston, USA; 3 Institute for Clinical Research and Health Policy Studies, Tufts Medical Center, Boston, MA USA; 4 Biostatistics, Epidemiology and Research Design (BERD) Center, Tufts Clinical and Translational Science Institute, Tufts University, Boston, MA USA; 5 Dialysis Clinic Incorporated, Nashville, TN USA

**Keywords:** ICH CAHPS, CAHPS, Dialysis, Patient experience

## Abstract

**Background:**

Patient experience in hemodialysis (HD) is measured twice yearly in all in-center HD patients in the United States using the In-Center Hemodialysis Consumer Assessment of Healthcare Providers and Systems (ICH CAHPS) survey. Survey scores are publically available and incorporated into the dialysis payment system. Despite its importance, little is known about factors associated with better experience scores. We studied the association between patient-level characteristics and experience scores in a large real-world cohort of HD patients.

**Methods:**

This is a cross-sectional analysis of ICH CAHPS administration in 2012. All in-center HD patients in Dialysis Clinic, Incorporated facilities nationally over 18 years old and receiving HD at their facility for at least 3 months were eligible. Predictors include patient demographic, clinical, and treatment-related characteristics. Outcomes include high global rating scores across three domains (Nephrologist, Dialysis Staff, Dialysis Center) and high composite scores across three domains (Nephrologists’ Communication and Caring, Quality of Dialysis Center Care and Operations, and Providing Information to Patients).

**Results:**

Among 3369 respondents, older age and telephone (vs. mail) administration of the survey were associated with higher global ratings, while shortened HD treatments were associated with lower global ratings. Lower education and telephone administration were associated with higher composite scores, while older age, and shortened HD treatments were associated with lower composite scores.

**Conclusions:**

Several patient characteristics and mode of survey administration are associated with higher experience scores. Future research should assess HD facility characteristics associated with higher scores and interventions that might improve experience accounting for these associations.

**Electronic supplementary material:**

The online version of this article (10.1186/s12882-018-1147-3) contains supplementary material, which is available to authorized users.

## Background

Dialysis patients comprise only 1% of the Medicare population, but account for 6 to 7% of Medicare costs [1]. To advance the Triple Aim [2] of improving patient experience, improving the health of populations, and reducing healthcare costs, the United States Centers for Medicare and Medicaid Services (CMS) instituted a value-based purchasing system called the End Stage Renal Disease (ESRD) Quality Incentive Program in 2012. This system set forth dialysis facility performance standards, the results of which are publicly reported and tied to payment penalties [3].

The Medicare Improvements for Patients and Providers Act of 2008 mandated that a quality metric should assess patient satisfaction [4]. Before this, CMS had begun development of a survey to assess hemodialysis (HD) patient experience; this work resulted in the In-Center Hemodialysis Consumer Assessment of Healthcare Providers and Systems (ICH CAHPS) survey, which was incorporated into the quality incentive program in 2014 [3]. The ICH CAHPS survey is part of a family of CAHPS patient experience surveys developed by the US Agency for Healthcare Research and Quality (AHRQ) to evaluate different parts of the healthcare system [5].

In its current form, the survey asks in-center HD patients 62 questions evaluating their experience with their nephrologist, dialysis staff, and dialysis facility [6]. Psychometric evaluation of this survey revealed adequate measures of validity and reliability for 2 of the 3 survey domains, including the domains assessing experience with the nephrologist and the dialysis staff [7, 8]. Mandatory biannual reporting for this measure started in 2016.

Little is known about what leads to better patient experience, as assessed by higher ICH CAHPS scores. Since 2014, regulation has barred US dialysis providers from obtaining patient-level survey results, making more detailed investigation of these relationships challenging. We performed a unique evaluation of the association between patient characteristics and ICH CAHPS survey scores using patient-level data from individuals treated at the largest not-for-profit dialysis provider in the United States, Dialysis Clinic Incorporated (DCI) in 2012.

## Methods

### Study population

All US in-center HD patients at least 18 years-old and treated at their facility for at least 3 months were eligible for the 2012 ICH CAHPS survey. HD providers identified vendors for survey administration according to AHRQ guidelines. Surveys were defined as ‘complete’ if at least 50% of predefined key questions were answered and if the patient reported receiving no assistance in survey completion. We included results from surveys administered August–October 2012 to all eligible HD patients from all DCI facilities across the US.

### Study design

We used patient characteristics data, as document in the DCI medical information system, to evaluate whether specific characteristics were associated with patient-level ICH CAHPS scores. A member of the DCI information technology team, who was independent from the research team, merged survey data with individual patient characteristics. De-identified data were subsequently sent to the authors. The study was approved by the Tufts Medical Center Investigational Review Board and underwent review by the DCI Administrative Review Office. DCI had signed a Respondent Identifiable Information Disclosure Agreement with the vendor, allowing DCI to receive the survey data exclusively for research purposes. This agreement predated the 2014 regulatory prohibition on reporting of patient-level data to dialysis providers.

### Survey

In 2012, ICH CAHPS was available in English and Spanish. The 2012 questionnaire included 58 questions that informed three composite scores and three global ratings (Additional file 1: Table S2). Composite scores for Nephrologists’ Communication and Caring (NCC), Quality of Dialysis Center Care and Operations (DCO), and Providing Information to Patients (PIP) were derived from questions with either yes/no or never/sometimes/usually/always responses (Table 1). Global ratings for the nephrologist, dialysis staff, and dialysis facility used a 10-point scale (0 being worst and 10 being best). The final result for each survey consisted of three composite and three global rating scores. Keeping with AHRQ scoring guidelines, we excluded surveys that did not fulfill the minimum key question requirement and those that indicated proxy help in survey completion (administration and management of the ICH CAHPS survey was transferred from AHRQ to CMS in 2014).

**Table 1:** Survey scoring domains and ‘primary study outcomes

Domain	Number of questions	Response options	Primary study outcome (‘Top Box’ outcome)
Nephrologists’ Communication and Caring (NCC)	6	Never/sometimes/usually/always (5) Yes/no (1)	Average equal to 4
Quality of Dialysis Center Care and Operations (DCO)	17	Never/sometimes/usually/always (14) Yes/no (3)	Average equal to 4
Providing Information to Patients (PIP)	9	Yes/no (9)	Average equal to 1
Nephrologist rating	1	0–10 (0 worst and 10 best)	9–10
Dialysis staff rating	1	0–10 (0 worst and 10 best)	9–10
Dialysis facility rating	1	0–10 (0 worst and 10 best)	9–10

### Survey administration

DCI provided its survey vendor with contact information for all patients who met eligibility criteria at the start of the survey period. About 10 days later, the vendor mailed a pre-notification letter informing patients of the upcoming survey and of its importance. The ICH CAHPS survey was mailed to patients the following week. Patients who did not respond within two weeks were sent a reminder letter, followed by another copy of the survey one month after the first survey. Patients were instructed to mail the completed survey directly back to the vendor. Up to three telephone calls were made over a 4-week period to invite non-responders to complete the survey by telephone. Dialysis facility staff were prohibited from any involvement, including discussing the survey with patients and caregivers.

### Covariates

Covariates were chosen a priori and included patient-level demographic, clinical and treatment characteristics collected routinely by DCI. Since the exact date of survey completion by each patient within the 3-month survey administration period is not known, all patient data (including demographics, clinical, and treatment characteristics) were obtained from the month the survey period started. Any missing data prompted a 3-month look back, from which the most recent value was used. Unexcused absence was defined as missing an entire HD treatment without rescheduling and without a reason such as hospitalization. Shortened treatment was defined as a delivered treatment that was at least 15 min shorter than prescribed. Hospitalization included hospital stays for any reason. Dialysis adequacy was described using urea clearance (expressed as spKt/V_urea_; CMS goal is > 1.2). Body mass index (BMI) was calculated using the estimated dry weight set by the patient’s nephrologist at the start of the survey period. ESRD vintage > 12 months before current facility was evaluated to identify patients new to a dialysis facility but having been on HD for at least 1 year, since patients who switch facilities might answer differently depending on the reason for switching.

### Outcomes

Global ratings and composite scores were converted into dichotomous outcomes based on whether or not each value fell within the “top box”, corresponding to CMS’ preferred responses [9]. In 2012 the top box for global ratings was a score of 8–10 which was subsequently changed to 9–10 in 2014. We used 9–10 to define top box for our primary analysis (Table 1), while sensitivity analyses were also performed using the 2012 top box definition (8–10) for global ratings (Additional file 1: Table S1). Composite scores are derived from a mix of questions that have either 2 level or 4 level responses, which are coded either ‘Yes = 1, No = 0′ for two-level responses and ‘Always=4, Usually=3, Sometimes=2, Never=1’ for four-level responses. The PIP composite contains all 2 level responses. The NCC and DCO composites have a mix of both types of questions; therefore, 2 level responses were recoded as Yes = 4 and No = 1 to facilitate calculation of the composite score in keeping with prior ARHQ recommendations. The top box for composite scores is defined as the highest attainable score after averaging responses to each question within a composite; we used an average equal to 4 for the NCC and DCO composites and 1 for the PIP composite (Table 1). Missing responses within a composite were handled using CMS’ current approach, which reduces the number of total questions in the denominator while calculating the average score. At least 50% of the questions within a composite had to be answered by the patient to trigger calculation of a composite score to obtain a reliable score.

### Statistical analysis

We used logistic regression models with random intercepts to account for possible clustering at the HD facility level. As is calculated by CMS, who reports all of the domains, study outcomes were attainment of top box score for each of the 3 global rating scores and 3 composite scores (6 separate outcomes). For the primary analysis we used patients with complete covariate data. A sensitivity analysis was performed using multiple imputation for missing covariate data (Additional file 1: Table S1) with models refitted and averaged using Rubin’s rule [10]. Since patients who responded by phone by definition were mail non-responders, we performed a sensitivity analysis using only mail responders. Given concern for multiple testing we used a Bonferroni corrected two-sided alpha of 0.01 to assess significance. All analyses were done using SAS Enterprise Guide (Version 7.12, Cary, NC) and R language (version 3.3.1, R Foundation for Statistical Computing, Vienna Austria).

## Results

### Study population

**Fig. 1:**
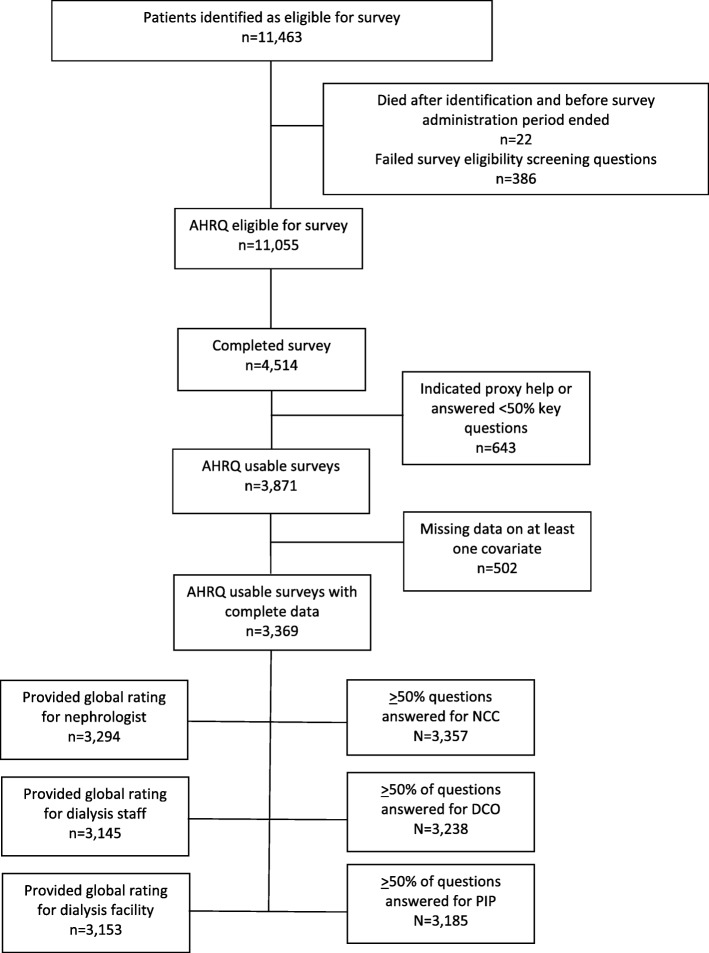
Flow Diagram. NCC: Nephrologists’ Communication and Caring DCO: Quality of Dialysis Center Care and Operations PIP: Providing Information to Patients

Of 11,055 eligible patients in 2012, 3871 (35%) responded and met criteria for completion of at least 50% of the key survey questions and independent completion of ICH CAHPS (Fig. 1). Patients were distributed across the country with good geographic representation (Additional file 1: Figure S1a and b). Of responders, 502 (13%) had missing data on at least one covariate and were excluded in primary analyses. Excluded patients were more often black and had shorter ESRD vintage (Table 2). Of the remaining 3369 patients, over 90% provided sufficient responses for at least one of the six outcomes. Within this population, mean age was 61 years, 46% were women, and 17% responded by telephone (Table 2 and Additional file 1: Table S3a-f). Median prescribed dialysis time was 3.5 h (interquartile range: 3.25, 4.0 h).

**Table 2:** Study population

	Population analyzed (*n* = 3369)	Excluded due to missing data^a^ (*n* = 502)
Age (years)	62.1 + 13.9	61.3 + 13.4
Female	1547 (45.9)	242 (48.2)
Race		
Black	1294 (38.4)	216 (48.0)
White	1917 (56.9)	212 (47.1)
Other	158 (4.7)	22 (4.9)
Hispanic Ethnicity	176 (5.2)	26 (6.0)
Cause of ESRD		
Diabetes	1357 (40.3)	193 (38.8)
Hypertension	960 (28.5)	150 (30.1)
Other	1052 (31.2)	155 (31.1)
Marital status		
Married	1465 (43.5)	160 (41.1)
Divorced/Separated	694 (20.6)	75 (19.3)
Widowed	476 (14.1)	68 (17.5)
Single	734 (21.8)	86 (22.1)
Education Level		
Grade School	271 (8.0)	33 (8.5)
High School	2082 (61.8)	221 (56.8)
College/Post Graduate	1016 (30.2)	135 (34.7)
English speaker	3326 (98.7)	373 (99.5)
Insurance		
Medicare/Medicaid	959 (28.5)	114 (25.9)
Medicare only	1533 (45.5)	226 (51.3)
Medicaid only	153 (4.5)	15 (3.4)
Other	724 (21.5)	86 (19.5)
Active on transplant waitlist	456 (13.5)	56 (16.5)
Vascular access		
Fistula	2198 (65.2)	322 (64.3)
Graft	703 (20.9)	95 (19.0)
Catheter	468 (13.9)	84 (16.8)
Albumin (g/dL)	3.9 + 0.4	3.9 ± 0.4
Hemoglobin (g/dL)	11.2 + 1.1	11.3 ± 1.2
Kt/V	1.63 + 0.27	1.59 ± 0.27
BMI (kg/m^2^)	29.2 + 7.6	29.5 ± 7.9
Unexcused absences	476 (14.1)	62 (12.4)
Treatments shortened	1481 (44.0)	221 (44.0)
Hospitalization	336 (10.0)	39 (7.8)
ESRD vintage (months)	37.6 (18.2, 72.1)	29.3 (13.3, 63.9)
ESRD vintage > 12 months before current facility	711 (21.1)	99 (19.7)
Ability to ambulate	2858 (84.8)	301 (80.3)
Ability to transfer	3047 (90.4)	321 (85.6)
Falls	312 (9.3)	29 (7.7)
ADL score	8 (5, 8)	8.0 (5.0, 8.0)
Response mode		
Mail	2800 (83%)	419 (83.5)
Telephone	569 (17%)	83 (16.5)

^a^Individuals excluded due to missing data on at least 1 covariate. Data presented as n (%), mean + standard deviation, or median (25th, 75th percentiles). *ESRD* End stage renal disease, *BMI* Body mass index, *ADL* Activities of daily living, Kt/V: Measure of dialysis adequacy. Unexcused absence was defined as missing an entire HD treatment without rescheduling; shortened treatment was defined as treatments that were at least 15 min shorter than prescribed; hospitalization included hospital stays for any reason

### Associations with global ratings

The distributions of global ratings were skewed with most responses being clustered towards the top (Additional file 1: Figure S2). In multivariable analyses, older age and telephone administration versus mail were associated with higher global ratings of nephrologists, dialysis staff, and dialysis facilities. Shortened treatments were associated with lower global ratings of nephrologists and dialysis facilities (Table 3).

**Table 3:** Multivariable association of characteristics with higher scores

	Nephrologist Rating (*N* = 3294)	Staff Rating (*N* = 3145)	Dialysis Facility Rating (*N* = 3153)	NCC Score (*N* = 3357)	DCO Score (*N* = 3238)	PIP Score (*N* = 3185)
ICC	0.07	0.09	0.07	0.03	0.06	0.03
Age, per 5 years	**1.06 (1.02, 1.11)**	**1.09 (1.04, 1.14)**	**1.10 (1.05, 1.15)**	1.01 (0.98, 1.05)	1.04 (0.99, 1.09)	**0.87 (0.84, 0.90)**
Female	1.00 (0.82, 1.21)	0.85 (0.68, 1.05)	0.82 (0.66, 1.02)	1.18 (1.00, 1.38)	0.89 (0.71, 1.12)	1.03 (0.87, 1.22)
Race, black vs white	1.06 (0.86, 1.31)	0.91 (0.72, 1.16)	0.88 (0.69, 1.11)	1.11 (0.93, 1.32)	0.87 (0.68, 1.12)	0.81 (0.67, 0.98)
Race, other vs white	0.72 (0.47, 1.11)	0.98 (0.59, 1.62)	0.93 (0.56, 1.56)	0.79 (0.54, 1.16)	0.64 (0.36, 1.13)	1.32 (0.90, 1.93)
Ethnicity, Hispanic vs non-Hispanic	1.19 (0.75, 1.88)	0.94 (0.56, 1.57)	1.19 (0.70, 2.04)	0.95 (0.64, 1.41)	1.19 (0.71, 1.97)	0.74 (0.49, 1.11)
Insurance, Medicare/Medicaid vs Medicare only	1.00 (0.79, 1.26)	0.97 (0.75, 1.25)	1.05 (0.81, 1.36)	1.07 (0.88, 1.30)	1.10 (0.84, 1.45)	0.91 (0.74, 1.12)
Insurance, Medicaid only vs Medicare only	0.99 (0.64, 1.53)	1.70 (1.00, 2.86)	1.16 (0.71, 1.90)	0.96 (0.65, 1.42)	1.24 (0.73, 2.10)	0.86 (0.58, 1.28)
Insurance, Other vs Medicare only	0.98 (0.78, 1.24)	0.94 (0.72, 1.23)	0.98 (0.75, 1.28)	1.06 (0.87, 1.29)	1.12 (0.86, 1.45)	0.91 (0.74, 1.12)
Marital status, married vs single	0.96 (0.75, 1.24)	1.12 (0.85, 1.48)	1.06 (0.80, 1.41)	1.22 (0.98, 1.53)	1.26 (0.92, 1.72)	1.21 (0.96, 1.52)
Marital status, divorced/separated vs single	0.97 (0.74, 1.26)	0.97 (0.72, 1.29)	0.94 (0.71, 1.26)	1.16 (0.92, 1.45)	1.08 (0.78, 1.50)	1.00 (0.79, 1.27)
Marital status, widowed vs single	1.18 (0.83, 1.69)	1.38 (0.92, 2.06)	1.32 (0.87, 2.00)	1.07 (0.80, 1.43)	1.16 (0.78, 1.73)	1.23 (0.90, 1.67)
Education level, grade school vs college or more	1.15 (0.79, 1.67)	1.19 (0.77, 1.84)	1.23 (0.79, 1.93)	1.14 (0.84, 1.54)	1.66 (1.12, 2.46)	1.18 (0.86, 1.63)
Education level, high school vs college or more	1.10 (0.91, 1.34)	1.13 (0.91, 1.41)	1.09 (0.88, 1.36)	1.10 (0.93, 1.29)	**1.41 (1.11, 1.78)**	1.09 (0.92, 1.30)
English speaker	0.20 (0.04, 0.87)	0.94 (0.33, 2.70)	0.85 (0.27, 2.69)	1.46 (0.69, 3.09)	1.76 (0.68, 4.59)	1.32 (0.62, 2.84)
Hospitalization	0.92 (0.69, 1.23)	0.98 (0.70, 1.37)	0.90 (0.64, 1.26)	1.03 (0.80, 1.32)	0.74 (0.51, 1.06)	1.00 (0.77, 1.31)
Active on transplant waitlist	1.23 (0.95, 1.61)	1.07 (0.80, 1.42)	0.91 (0.69, 1.21)	1.24 (1.00, 1.55)	0.68 (0.48, 0.95)	**1.36 (1.08, 1.70)**
BMI, per 2 kg/m^2^	1.02 (1.00, 1.05)	1.01 (0.98, 1.04)	1.02 (0.99, 1.05)	1.00 (0.98, 1.02)	0.97 (0.95, 1.00)	1.02 (0.99, 1.04)
Cause ESRD, diabetes vs. other	0.88 (0.71, 1.09)	1.02 (0.79, 1.30)	0.98 (0.77, 1.26)	0.92 (0.77, 1.11)	0.93 (0.72, 1.19)	1.05 (0.87, 1.28)
Cause ESRD, hypertension vs. other	0.95 (0.75, 1.20)	1.09 (0.84, 1.42)	0.99 (0.76, 1.29)	0.93 (0.77, 1.14)	1.03 (0.79, 1.34)	1.20 (0.98, 1.48)
Vascular access, catheter vs. fistula	0.91 (0.70, 1.18)	0.87 (0.65, 1.17)	0.90 (0.67, 1.21)	0.90 (0.72, 1.13)	1.32 (0.99, 1.75)	1.00 (0.79, 1.26)
Vascular access, graft vs. fistula	1.00 (0.79, 1.25)	1.11 (0.86, 1.44)	1.21 (0.93, 1.57)	0.92 (0.77, 1.11)	0.98 (0.75, 1.27)	1.18 (0.97, 1.44)
Hemoglobin, per 0.5 g/dL	0.95 (0.92, 0.99)	0.98 (0.94, 1.03)	0.97 (0.93, 1.01)	1.00 (0.96, 1.03)	0.99 (0.95, 1.04)	0.99 (0.96, 1.03)
Albumin, per 0.2 g/dL	0.99 (0.94, 1.04)	0.95 (0.89, 1.00)	0.94 (0.89, 1.00)	1.01 (0.97, 1.06)	1.03 (0.97, 1.09)	1.03 (0.99, 1.08)
Kt/V, per 0.2	1.03 (0.96, 1.11)	1.04 (0.96, 1.13)	1.09 (1.00, 1.18)	**1.09 (1.02, 1.15)**	1.06 (0.98, 1.15)	0.95 (0.89, 1.02)
ESRD vintage, per 12 months	1.01 (0.98, 1.05)	0.97 (0.93, 1.01)	0.97 (0.93, 1.00)	1.00 (0.97, 1.03)	0.95 (0.92, 0.99)	0.99 (0.96, 1.02)
ESRD vintage > 12 months before current facility	0.91 (0.71, 1.16)	0.90 (0.69, 1.18)	1.05 (0.80, 1.39)	1.15 (0.93, 1.41)	0.79 (0.58, 1.08)	0.86 (0.69, 1.07)
Unexcused absences	0.82 (0.64, 1.04)	0.84 (0.64, 1.09)	0.84 (0.64, 1.10)	0.95 (0.76, 1.17)	0.69 (0.50, 0.96)	0.86 (0.69, 1.08)
Treatments shortened	**0.71 (0.59, 0.85)**	**0.77 (0.63, 0.95)**	**0.74 (0.60, 0.91)**	**0.75 (0.64, 0.87)**	0.90 (0.73, 1.11)	0.87 (0.74, 1.02)
Telephone administration vs mail	**1.51 (1.17, 1.94)**	**1.70 (1.28, 2.27)**	**1.91 (1.42, 2.58)**	1.21 (0.99, 1.48)	**1.96 (1.53, 2.51)**	**1.43 (1.17, 1.75)**

Data shown as odds ratio (OR) (95% CI) adjusted for all other variables in the table. Odds ratio above 1.00 is associated with top box response. Associations with *p* < 0.01 are in bold. *NCC* Nephrologists’ Communication and Caring, *DCO* Quality of Dialysis Center Care and Operations, *PIP* Providing Information to Patients, *ESRD* End stage renal disease, *BMI* Body mass index; Kt/V: Measure of dialysis adequacy. Unexcused absence was defined as missing an entire HD treatment without rescheduling; shortened treatment was defined as treatments that were at least 15 min shorter than prescribed; hospitalization included hospital stays for any reason

### Associations with composite scores

The distributions of composite scores were also skewed with most responses clustered at the top end (Additional file 1: Figure S2). Higher dialysis clearance (expressed as spKt/V_urea_) was associated with higher scores for Nephrologists’ Communication and Caring (NCC) in multivariable analyses, whereas shortened treatments were associated with lower scores. Lower educational level and telephone administration were significantly associated with higher Quality of Dialysis Center Care and Operations (DCO) scores. Lastly, being active on the kidney transplant list and telephone administration were significantly associated with higher scores for Providing Information to Patients (PIP), while older age was associated with lower PIP scores (Table 3).

### Sensitivity analyses

Among the 502 (13%) individuals with missing covariate data, most missing data were on functional covariates, which were used for exploratory analyses only. Overall results were similar after multiple imputation for missing covariates (Additional file 1: Table S4), after changing the global rating top box score to 8–10 to be consistent the scoring methodology used prior to 2014 (Additional file 1: Table S5), and after removing telephone responders from the analysis (Additional file 1: Table S6). Additionally, intraclass coefficients were very low in each model making clustering of results at the facility level less likely.

## Discussion

In this national sample of in-center hemodialysis respondents to the ICH CAHPS survey, older age and telephone administration of the survey were consistently associated with higher global ratings, while shortened treatments were associated with lower global ratings for self-reported patient experience. Telephone administration of the survey was consistently associated with higher composite scores for self-reported patient experience. Other factors like older age, kidney transplant listing and shortened treatments were variably associated with self-reported patient experience depending on whether facility quality and operations, nephrologists’ communication and caring, or provision of information were being assessed, showing that patients differentiate their experience dependent on the composite area being evaluated.

Prior literature examining patient-level characteristics associated with ICH CAHPS scores consists of only one small study in which 404 patients, selected by nephrologists, self-reported their demographic and clinical characteristics [8]. In unadjusted univariate analyses, black race was associated with lower dialysis facility global ratings. Another study of patient-level Medicare-CAHPS responses from a group of dialysis patients (a non-dialysis specific survey in use prior to ICH CAHPS) showed that self-reported black race and lower education were associated with lower rating of care and with lower physician communication scores [11]. Several recent studies have evaluated the association between facility characteristics and ICH CAHPS scores. In one, a higher proportion of Black and Native American patient populations, for-profit dialysis facility status, and large dialysis organization status were associated with lower survey scores, while having a larger proportion of privately insured patients, smaller facility size, and more nurses per patient were associated with higher scores [12]. A second recent study of facility-level ICH CAHPS scores showed association between high Quality Incentive Program scores and most of the ICH CAHPS survey domains [13, 14]. Our evaluation of patient satisfaction at DCI facilities in 2011, using an internally developed DCI survey, showed white race, older age, shorter dialysis vintage, fewer shortened treatments and fewer missed treatments to be associated with higher scores [15]. Lastly, in one of the only international studies using a different questionnaire across centers in Europe and South America, older age was associated with higher experience scores while presence of depressive symptoms was associated with lower scores [16].

Most care provided during in-center hemodialysis in the United States is delivered by patient care technicians along with hemodialysis nurses. Dieticians and social workers are also present in US dialysis facilities. Ratios differ by state, but 3 patients per technician or nurse is common and 1 social worker and dietician per 75 to 100 patients is also common [17]. Patients are generally seen by nephrologists during their dialysis session 1 to 4 times, although contact time between patients and physicians can vary from exceptionally brief to prolonged. Although highly variable across dialysis facilities, in general, patients spend more time interacting with nurses, technicians, social workers, and dieticians than nephrologists.

In our analyses, we found that demographic characteristics associated with ICH CAHPS scores included age and educational level. Older age was consistently associated with higher global ratings for nephrologists, dialysis staff, and dialysis facilities but with a lower PIP composite score. Since questions comprising the PIP composite rely more on recall of information or on patient teaching than other questions, this association may reflect the increased prevalence of cognitive impairment among older dialysis patients [18–20]. It is possible that older patients would benefit from receiving dialysis related information differently, perhaps in smaller chunks reinforced over an extended period of time. For reasons that are not readily apparent, lower education was associated with higher DCO composite score. Counter-intuitively, education level was not associated with the PIP score. Qualitative research examining attitudes towards nephrologists, dialysis staff, and dialysis facilities may generate hypotheses to explain lower global rating scores among younger patients.

Clinical characteristics associated with ICH CAHPS scores include being active on the kidney transplant list and shortened treatments. Being active on the kidney transplant list was associated with higher PIP composite scores. This could be a reflection of the added communication through kidney transplant clinic visits and additional discussions that these patients have with caregivers compared to those who are inactive or ineligible for kidney transplant. Shortened treatments were associated with lower NCC composite scores. This result with respect to adherence raises the possibility of a bidirectional, if not cyclical, relationship, whereby poor care experience leads to poor adherence, while physician and dialysis facility staff reactions to non-adherence could strain the relationship and worsen the care experience. It also may suggest other common factors linking worse adherence and worse experience such as unaddressed pain, depression and lower health literacy [21, 22]. These associations suggest that interventions for such patients could result in substantial benefit, particularly in view of the association of worse adherence with an increased risk of adverse patient outcomes, including death [23, 24].

Finally, telephone rather than mail administration was associated with higher scores on all three global rating scales and on all of the composite scores except for NCC. This finding is consistent with prior literature showing more positive responses to CAHPS surveys when they were administered over the telephone rather than by mail [25–27]. This finding is important, because, although dialysis providers are able to choose which mode of administration is used by their vendor for this survey, telephone administration adds substantially to the cost of survey administration.

In view of the influence of patient characteristics on ICH CAHPS scores, CMS in 2015 began to use internal monitoring data to adjust facility scores on the basis of survey administration mode and a limited number of patient-reported characteristics [9]. This adjustment changes yearly and is not subject to external review. Current CMS adjustment uses a limited number of patient-reported characteristics, some of which overlap with ones we found to be significantly associated with scores (including age, mode of survey administration, and education level). CMS does not adjust for clinical characteristics that vary among facilities such as kidney transplant eligibility, socioeconomic status or treatment adherence.

Our study has several strengths, including multivariable analysis of patient-level ICH CAHPS survey responses from a large national sample of HD patients, using detailed clinical and demographic information collected by the dialysis facility. No previous literature describes the characteristics associated with higher ICH CAHPS survey scores after multivariable adjustment. Because, beginning in 2014, CMS barred dialysis facilities from obtaining patient-level ICH CAHPS survey results, such an analysis is possible only using data collected in 2012 and 2013 (prior to ICH CAHPS incorporation into the ESRD Quality Incentive Program). To the best of our knowledge, DCI is the only dialysis provider to obtain these patient-level survey results prior to the regulatory prohibition, making this endeavor unique. We show robust results across models and several sensitivity analyses including one utilizing the older AHRQ top box definition that was in use until 2013.

Limitations to this study include missing data and application of CMS’ facility-level scoring method to our patient-level survey results. As with other survey data, low response rates [28] raise concern for non-response bias. In our analyses we perform multiple testing in an attempt to mirror the way CMS currently scores these surveys as well as use a Bonferroni corrected *p*-value to try and limit the false positive findings.

Patient experience surveys are a vital part of any value-based purchasing model to ensure quality of care. Our findings are particularly relevant because surveys completed from 2016 onwards are tied to dialysis facility reimbursements and are projected to assume greater weight in coming years in this pay-for-performance system [29]. Additionally, the breadth of these surveys will increase pending the development of a home dialysis CAHPS version. With this increasing prominence and absence of interventions shown to improve scores, our findings lay the groundwork for dialysis providers to enhance efforts to understand drivers of better HD experience. This work is timely since, despite survey use for more than four years, proven strategies to address low experience scores are uncertain. This work is also unique since regulatory prohibition does not allow patient-level survey data to be obtained since 2014. Our work combined with further qualitative work with dialysis patients will help elucidate possible interventions that could improve experience scores moving forward.

## Conclusions

Several patient characteristics and mode of survey administration are associated with higher HD experience scores as measured by the ICH CAHPS survey. These findings are very important since this survey is relatively new and survey scores carry important financial and policy implications. Future research should assess HD facility-level characteristics associated with higher scores and interventions that might improve experience.

## Additional files

Additional file 1: **Table S1.** Analyses performed. **Table S2.** ICH CAHPS questions used for scoring in 2012. **Figure S1A.** Geographic distribution of DCI clinics. **B** Geographic distribution of responders. **Figure S2.** Distribution of scores. **Table S3A.** Patient Characteristics stratified by higher or lower nephrologist rating. B Patient Characteristics stratified by higher or lower dialysis staff rating. C Patient Characteristics stratified by higher or lower dialysis facility rating. D Patient Characteristics stratified by higher or lower Nephrologists’ Communication and Caring (NCC) score. E Patient Characteristics stratified by higher or lower Quality of Dialysis Center Care and Operations (DCO) score. F Patient Characteristics stratified by higher or lower Providing Information to Patients (PIP) score. **Table S4A.** Multivariable association of characteristics with higher nephrologist rating with multiple imputation. B Multivariable association of characteristics with higher dialysis staff rating with multiple imputation. C Multivariable association of characteristics with higher dialysis facility rating with multiple imputation. D Multivariable association of characteristics with higher Nephrologists’ Communication and Caring (NCC) score with multiple imputation. E Multivariable association of characteristics with higher Quality of Dialysis Center Care and Operations (DCO) score with multiple imputation. F Multivariable association of characteristics with higher Providing Information to Patients (PIP) score with multiple imputation. **Table S5A.** Multivariable association of characteristics with higher nephrologist rating using older top box definition. B Multivariable association of characteristics with higher dialysis staff rating using older top box definition. C Multivariable association of characteristics with higher dialysis facility rating using older top box definition. **Table S6.** Multivariable association of characteristics with higher scores after excluding patients who responded by phone. (PDF 452 kb)

## Abbreviations

AHRQ: Agency for Healthcare Research and Quality
BMI: Body mass index
CMS: Centers for Medicare and Medicaid Services
DCI: Dialysis Clinic Incorporated
DCO: Quality of Dialysis Center Care and Operations
ESRD: End-stage renal disease
HD: Hemodialysis
ICH CAHPS: In-Center Hemodialysis Consumer Assessment of Healthcare Providers and Systems
NCC: Nephrologists’ Communication and Caring
PIP: Providing Information to Patients
US: United States
