# Supplementary material for: Hemodialysis patient characteristics associated with better experience as measured by the In-center Hemodialysis Consumer Assessment of Healthcare Providers and Systems (ICH CAHPS) survey
Source: BMC Nephrol. 2018 Nov 28;19:340. doi: 10.1186/s12882-018-1147-3 (PMC6264620; doi:10.1186/s12882-018-1147-3)
Supplement: Supplementary file 1 — Table S1. Analyses performed. Table S2. ICH CAHPS questions used for scoring in 2012. Figure S1A. Geographic distribution of DCI clinics. B Geographic distribution of responders. Figure S2. Distribution of scores. Table S3A. Patient Characteristics stratified by higher or lower nephrologist rating. B Patient Characteristics stratified by higher or lower dialysis staff rating. C Patient Characteristics stratified by higher or lower dialysis facility rating. D Patient Characteristics stratified by higher or lower Nephrologists’ Communication and Caring (NCC) score. E Patient Characteristics stratified by higher or lower Quality of Dialysis Center Care and Operations (DCO) score. F Patient Characteristics stratified by higher or lower Providing Information to Patients (PIP) score. Table S4A. Multivariable association of characteristics with higher nephrologist rating with multiple imputation. B Multivariable association of characteristics with higher dialysis staff rating with multiple imputation. C Multivariable association of characteristics with higher dialysis facility rating with multiple imputation. D Multivariable association of characteristics with higher Nephrologists’ Communication and Caring (NCC) score with multiple imputation. E Multivariable association of characteristics with higher Quality of Dialysis Center Care and Operations (DCO) score with multiple imputation. F Multivariable association of characteristics with higher Providing Information to Patients (PIP) score with multiple imputation. Table S5A. Multivariable association of characteristics with higher nephrologist rating using older top box definition. B Multivariable association of characteristics with higher dialysis staff rating using older top box definition. C Multivariable association of characteristics with higher dialysis facility rating using older top box definition. Table S6. Multivariable association of characteristics with higher scores after excluding patients who responded by pho [file 12882_2018_1147_MOESM1_ESM.pdf]

Supplementary information:

Table 1: Analyses performed

	**Primary analysis**	**Sensitivity analysis 1**	**Sensitivity analysis 2**	**Sensitivity analysis 3**
**Top box definition for global ratings**	9-10	9-10	8-10	8-10
**Top box definition for composite scores**	Average equal to 4 for NCC and DCO and 1 for PIP	Average equal to 4 for NCC and DCO and 1 for PIP	Average equal to 4 for NCC and DCO and 1 for PIP	Average equal to 4 for NCC and DCO and 1 for PIP
**Missing covariate data**	Not included	Multiple imputation performed	Not included	Multiple imputation performed
**Incomplete composite responses**	Decreased denominator to obtain average*	Decreased denominator to obtain average*	Decreased denominator to obtain average*	Decreased denominator to obtain average*
**<50% key questions answered**	Not included	Not included	Not included	Not included
**Proxy help indicated**	Not included	Not included	Not included	Not included

*As long as at least 50% of the questions within a composite were answered otherwise individual was not included. 9-10 is the current CMS top box definition and 8-10 was the older AHRQ top box definition. NCC: Nephrologists' Communication and Caring; DCO: Quality of Dialysis Center Care and Operations; PIP: Providing Information to Patients

Table 2: ICH CAHPS questions used for scoring in 2012

Question	Nephrologists' Communication and Caring (NCC)
3	In the last 3 months, how often did your kidney doctors listen carefully to you?
4	In the last 3 months, how often did your kidney doctors explain things in a way that was easy to understand?
5	In the last 3 months, how often did your kidney doctors show respect for what you had to say?
6	In the last 3 months, how often did your kidney doctors spend enough time with you?
7	In the last 3 months, how often did you feel your kidney doctors really cared about you as a person?
9	Do your kidney doctors seem informed and up to date about the health care you receive from other doctors?
	**Quality of Dialysis Center Care and Operations (DCO)**
10	In the last 3 months, how often did the dialysis center staff listen carefully to you?
11	In the last 3 months, how often did the dialysis center staff explain things in a way that was easy to understand?
12	In the last 3 months, how often did the dialysis center staff show respect for what you had to say?
13	In the last 3 months, how often did the dialysis center staff spend enough time with you?
14	In the last 3 months, how often did you feel the dialysis center staff really cared about you as a person?
15	In the last 3 months, how often did the dialysis center staff make you as comfortable as possible during dialysis?
16	In the last 3 months, did dialysis center staff keep information about you and your health as private as possible from other patients?
17	In the last 3 months, did you feel comfortable asking dialysis center staff everything you wanted about dialysis care?
21	In the last 3 months, how often did dialysis center staff insert your needles with as little pain as possible?
22	In the last 3 months, how often did dialysis center staff check you as closely as you wanted while you were on the dialysis machine?
24	In the last 3 months, how often were the dialysis center staff able to manage problems during your dialysis?
25	In the last 3 months, how often did dialysis center staff behave in a professional manner?
26	In the last 3 months, did dialysis center staff talk to you about what you should eat and drink?
27	In the last 3 months, how often did dialysis center staff explain blood test results in a way that was easy to understand?
33	In the last 3 months, when you arrived on time, how often did you get put on the dialysis machine within 15 minutes of your appointment or shift time?
34	In the last 3 months, how often was the dialysis center as clean as it could be?
43	In the last 12 months, how often were you satisfied with the way they handled these problems?
	**Providing Information to Patients (PIP)**
19	Do you know how to take care of your graft, fistula, or catheter?
28	Did this dialysis center ever give you any written information about your rights as a patient?

28	Did dialysis center staff at this center ever review your rights as a patient with you?
30	Have dialysis center staff ever told you what to do if you experience a health problem at home?
31	Have any dialysis center staff ever told you how to get off the machine if there is an emergency at the center?
36	In the last 12 months, did either your kidney doctors or dialysis center staff talk to you as much as you wanted about which treatment is right for you?
38	In the last 12 months, have either your kidney doctors or dialysis center staff explained to you why you are not eligible for a kidney transplant?
39	In the last 12 months, did either your kidney doctors or dialysis center staff talk to you about peritoneal dialysis?
40	In the last 12 months, were you as involved as much as you wanted in choosing the treatment for kidney disease that is right for you?

Figure 1a: Geographic distribution of DCI clinics

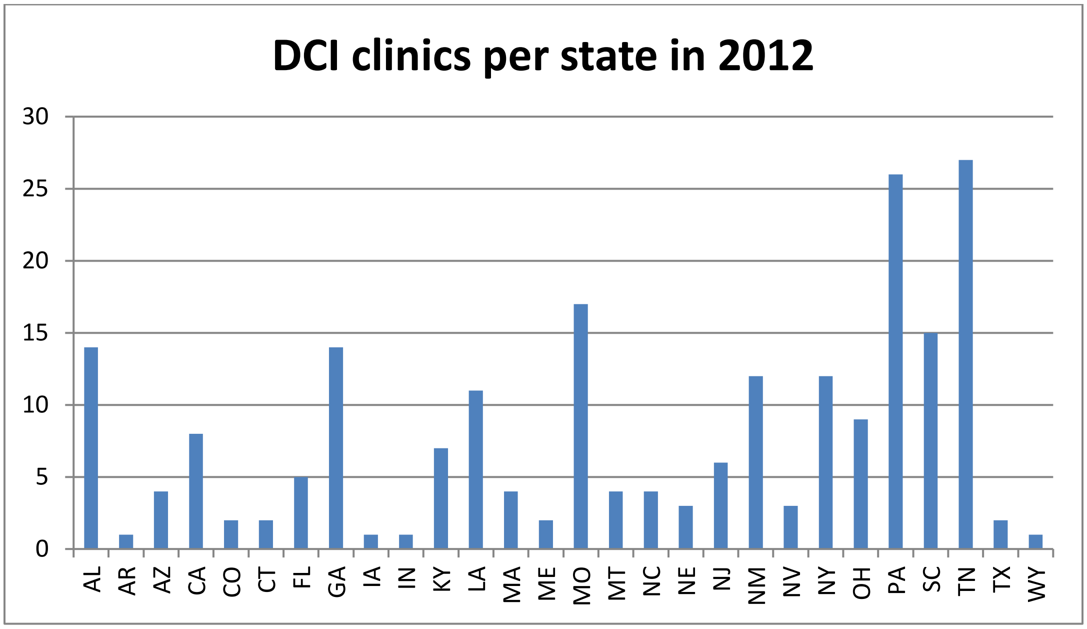

Figure 1b: Geographic distribution of responders

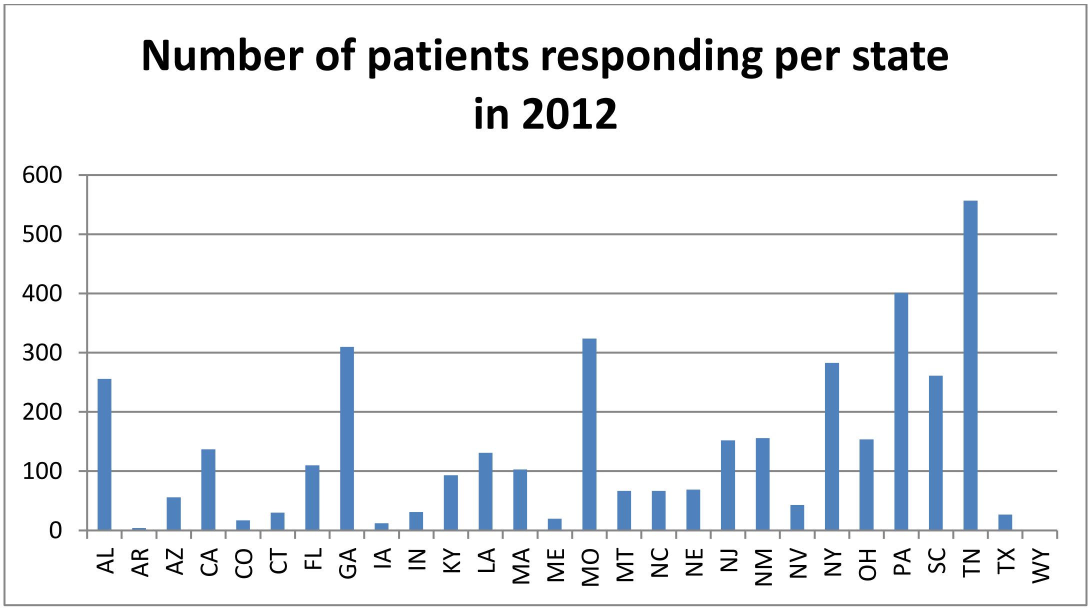

Figure 2: Distribution of scores

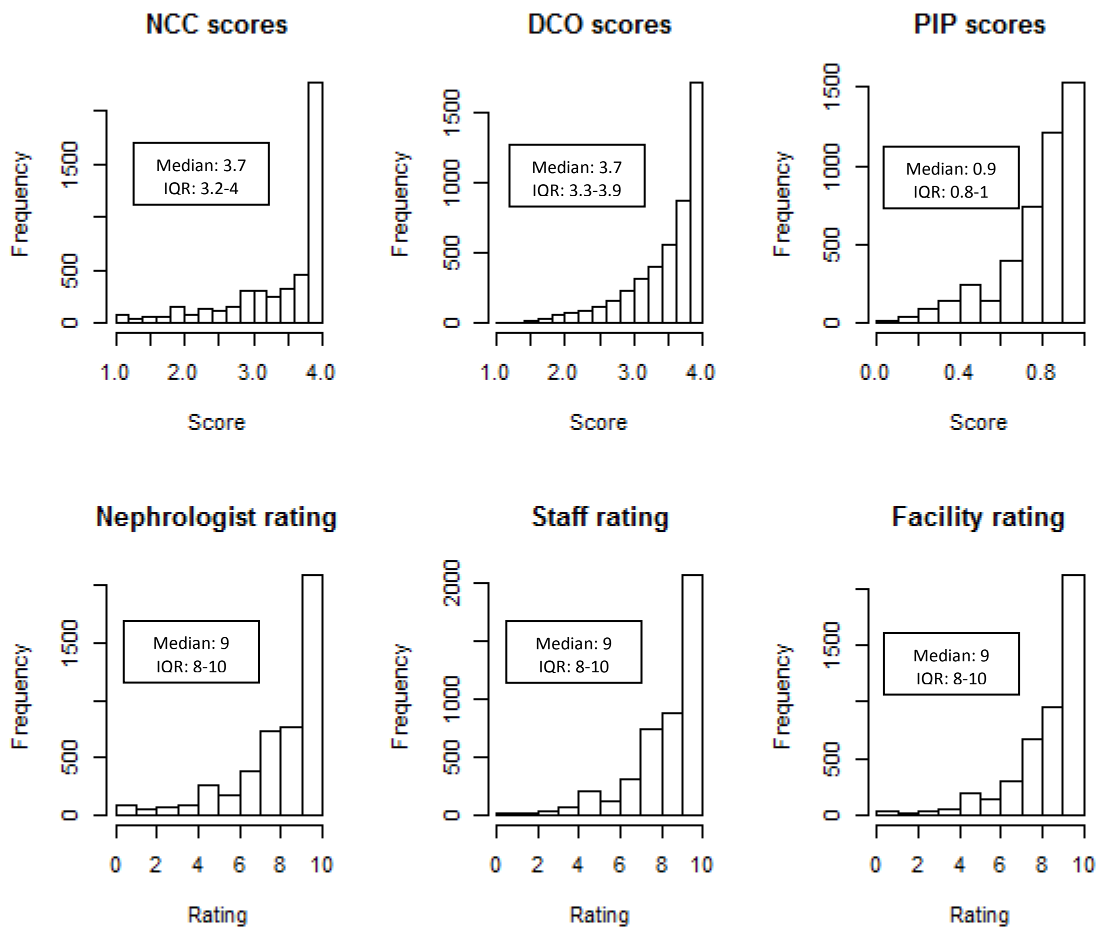

Table 3a: Patient Characteristics stratified by higher or lower nephrologist rating

	Total (n=3,294)	Top Box (n=2,045, 62%)	Below Top Box (n=1249, 38%)
Age (years)	61.9 ± 13.9	63.0 ± 13.7	60.2 ± 13.9
Female	1498 (45.5)	978 (47.8)	520 (41.6)
Race			
Black	1269 (38.5)	800 (39.1)	469 (37.6)
White	1870 (56.8)	1161 (56.8)	709 (56.8)
Other	155 (4.7)	84 (4.1)	71 (5.7)
Hispanic Ethnicity	171 (5.2)	103 (5.0)	68 (5.4)
Cause of ESRD			
Diabetes	1322 (40.1)	795 (38.9)	527 (42.2)
Hypertension	939 (28.5)	603 (29.5)	336 (26.9)
Other	1033 (31.4)	647 (31.6)	386 (30.9)
Marital status			
Married	1437 (43.6)	909 (44.5)	528 (42.3)
Divorced/Separated	676 (20.5)	409 (20.0)	267 (21.4)
Widowed	455 (13.8)	315 (15.4)	140 (11.2)
Single	726 (22.0)	412 (20.2)	314 (25.1)
Education Level			
Grade School	258 (7.8)	173 (8.5)	85 (6.8)
High School	2039 (61.9)	1285 (62.8)	754 (60.4)
College/Post Graduate	997 (30.3)	587 (28.7)	410 (32.8)
English speaker	3254 (98.8)	2014 (98.5)	1240 (99.3)
Insurance			
Medicare/Medicaid	930 (28.2)	561 (27.4)	369 (29.5)
Medicare only	1507 (45.8)	953 (46.6)	554 (44.4)
Medicaid only	148 (4.5)	83 (4.1)	65 (5.2)
Other	709 (21.5)	448 (21.9)	261 (20.9)
Active on transplant waitlist	454 (13.8)	294 (14.4)	160 (12.8)
Vascular access			
Fistula	2152 (65.3)	1336 (65.3)	816 (65.3)
Graft	456 (13.8)	440 (21.5)	246 (19.7)
Catheter	686 (20.8)	269 (13.2)	187 (15.0)
Albumin (g/dL)	3.9 ± 0.4	3.88 ± 0.4	3.89 ± 0.4
Hemoglobin (g/dL)	11.2 ± 1.1	11.2 ± 1.1	11.3 ± 1.2
Kt/V	1.62 ± 0.27	1.64 ± 0.27	1.60 ± 0.27
BMI (kg/m^2)	29.2 ± 7.7	29.1 ± 7.5	29.3 ± 7.8
Unexcused absences	463 (14.1)	250 (12.2)	213 (17.1)
Treatments shortened	1445 (43.9)	811 (39.7)	634 (50.8)
Hospitalization	331 (10.1)	197 (9.6)	134 (10.7)
ESRD vintage (months)	37.8 (18.2, 72.3)	37.1 (17.7, 71.1)	39.1 (19.2, 73.7)
ESRD vintage > 12 months before current facility	695 (21.1)	414 (20.2)	281 (22.5)

Ability to ambulate	2800 (85.0)	1734 (84.8)	1066 (85.4)
Ability to transfer	2981 (90.5)	1843 (90.1)	1138 (91.1)
Falls	301 (9.1)	176 (8.6)	125 (10.0)
ADL score	8 (5, 8)	8 (5, 8)	8 (5, 8)
Response mode			
Mail	2739 (83.2)	1690 (82.6)	1049 (84.0)
Telephone	555 (16.9)	355 (17.4)	200 (16.0)

Top box score defined as 9-10 on the global rating scale. Data presented as n (%), mean $\pm$ standard deviation, or median (25th, 75th percentiles). ESRD: End stage renal disease; BMI: Body mass index; ADL: Activities of daily living; Kt/V: Measure of dialysis adequacy. Unexcused absence was defined as missing an entire HD treatment without rescheduling; shortened treatment was defined as treatments that were at least 15 minutes shorter than prescribed; hospitalization included hospital stays for any reason.

Table 3b: Patient Characteristics stratified by higher or lower dialysis staff rating

	Total (n=3,145)	Top Box (n=2,071, 66%)	Below Top Box (n=1,074, 34%)
Age (years)	61.8 ± 13.9	62.8 ± 13.6	59.8 ± 14.2
Female	1433 (45.6)	949 (45.8)	484 (45.1)
Race			
Black	1201 (38.2)	751 (36.3)	450 (41.9)
White	1795 (57.1)	1226 (59.2)	569 (53.0)
Other	149 (4.7)	94 (4.5)	55 (5.1)
Hispanic Ethnicity	173 (5.5)	108 (5.2)	65 (6.1)
Cause of ESRD			
Diabetes	1253 (39.8)	838 (40.5)	415 (38.6)
Hypertension	891 (28.3)	589 (28.4)	302 (28.1)
Other	1001 (31.8)	644 (31.1)	357 (33.2)
Marital status			
Married	1370 (43.6)	921 (44.5)	449 (41.8)
Divorced/Separated	641 (20.4)	409 (19.8)	232 (21.6)
Widowed	427 (13.6)	311 (15.0)	116 (10.8)
Single	707 (22.5)	430 (20.8)	277 (25.8)
Education Level			
Grade School	256 (8.1)	187 (9.0)	69 (6.4)
High School	1946 (61.9)	1315 (63.5)	631 (58.8)
College/Post Graduate	943 (30.0)	569 (27.5)	374 (34.8)
English speaker	3103 (98.7)	2043 (98.7)	1060 (98.7)
Insurance			
Medicare/Medicaid	903 (28.7)	568 (27.4)	335 (31.2)
Medicare only	1424 (45.3)	958 (46.3)	466 (43.4)
Medicaid only	143 (4.6)	99 (4.8)	44 (4.1)
Other	675 (21.5)	446 (21.5)	229 (21.3)
Active on transplant waitlist	434 (13.8)	277 (13.4)	157 (14.6)
Vascular access			
Fistula	2064 (65.6)	1352 (65.3)	712 (66.3)
Graft	436 (13.9)	449 (21.7)	196 (18.3)
Catheter	645 (20.5)	270 (13.0)	166 (15.5)
Albumin (g/dL)	3.9 ± 0.4	3.9 ± 0.4	3.9 ± 0.4
Hemoglobin (g/dL)	11.2 ± 1.1	11.2 ± 1.1	11.2 ± 1.2
Kt/V	1.62 ± 0.27	1.63 ± 0.26	1.61 ± 0.29
BMI (kg/m^2)	29.2 ± 7.7	29.2 ± 7.6	29.1 ± 7.7
Unexcused absences	454 (14.4)	282 (13.6)	172 (16.0)
Treatments shortened	1381 (43.9)	853 (41.2)	528 (49.2)
Hospitalization	313 (10.0)	205 (9.9)	108 (10.1)
ESRD vintage (months)	38.1 (18.4, 72.5)	36.9 (17.9, 68.5)	40.3 (19.3, 77.5)
ESRD vintage > 12 months before current facility	670 (21.3)	411 (19.9)	259 (24.1)

Ability to ambulate	2681 (85.3)	1760 (85.0)	921 (85.8)
Ability to transfer	2853 (90.7)	1882 (90.9)	971 (90.4)
Falls	284 (9.0)	187 (9.0)	97 (9.0)
ADL score	8 (5, 8)	6.5 ± 2.1	6.5 ± 2.1
Response mode			
Mail	2589 (82.3)	1686 (81.4)	903 (84.1)
Telephone	556 (17.7)	385 (18.6)	171 (15.9)

Top box score defined as 9-10 on the global rating scale. Data presented as n (%), mean ± standard deviation, or median (25th, 75th percentiles). ESRD: End stage renal disease; BMI: Body mass index; ADL: Activities of daily living; Kt/V: Measure of dialysis adequacy. Unexcused absence was defined as missing an entire HD treatment without rescheduling; shortened treatment was defined as treatments that were at least 15 minutes shorter than prescribed; hospitalization included hospital stays for any reason.

Table 3c: Patient Characteristics stratified by higher or lower dialysis facility rating

	Total (n=3,153)	Top Box (n=2,157, 68%)	Below Top Box (n=996, 32%)
Age (years)	61.8 ± 13.9	62.9 ± 13.5	59.4 ± 14.2
Female	1432 (45.4)	983 (45.6)	449 (45.1)
Race			
Black	1200 (38.1)	787 (36.5)	413 (41.5)
White	1804 (57.2)	1268 (58.8)	536 (53.8)
Other	149 (4.7)	102 (4.7)	47 (4.7)
Hispanic Ethnicity	174 (5.5)	121 (5.6)	53 (5.3)
Cause of ESRD			
Diabetes	1252 (39.7)	875 (40.6)	377 (37.9)
Hypertension	897 (28.5)	613 (28.4)	284 (28.5)
Other	1004 (31.8)	669 (31.0)	335 (33.6)
Marital status			
Married	1376 (43.6)	968 (44.9)	408 (41.0)
Divorced/Separated	644 (20.4)	432 (20.0)	212 (21.3)
Widowed	428 (13.6)	322 (14.9)	106 (10.6)
Single	705 (22.4)	435 (20.2)	270 (27.1)
Education Level			
Grade School	258 (8.2)	203 (9.4)	55 (5.5)
High School	1949 (61.8)	1360 (63.1)	589 (59.1)
College/Post Graduate	946 (30.0)	594 (27.5)	352 (35.3)
English speaker	3110 (98.6)	2119 (98.2)	991 (99.5)
Insurance			
Medicare/Medicaid	907 (28.8)	596 (27.6)	311 (31.2)
Medicare only	1426 (45.2)	1002 (46.5)	424 (42.6)
Medicaid only	142 (4.5)	94 (4.4)	48 (4.8)
Other	678 (21.5)	465 (21.6)	213 (21.4)
Active on transplant waitlist	435 (13.8)	282 (13.1)	153 (15.4)
Vascular access			
Fistula	2070 (65.7)	1396 (64.7)	674 (67.7)
Graft	434 (13.8)	460 (21.3)	189 (19.0)
Catheter	649 (20.6)	301 (14.0)	133 (13.4)
Albumin (g/dL)	3.9 ± 0.4	3.9 ± 0.4	3.9 ± 0.4
Hemoglobin (g/dL)	11.2 ± 1.2	11.2 ± 1.1	11.2 ± 1.2
Kt/V	1.62 ± 0.27	1.63 ± 0.26	1.62 ± 0.29
BMI (kg/m^2)	29.2 ± 7.7	29.2 ± 7.7	29.1 ± 7.6
Unexcused absences	455 (14.4)	288 (13.4)	167 (16.8)
Treatments shortened	1383 (43.9)	891 (41.3)	492 (49.4)
Hospitalization	315 (10.0)	209 (9.7)	106 (10.6)
ESRD vintage (months)	38.0 (18.3, 72.4)	36.4 (17.5, 68.5)	41.4 (21.8, 77.4)
ESRD vintage > 12 months before current facility	671 (21.3)	424 (19.7)	247 (24.8)

Ability to ambulate	2690 (85.3)	1831 (84.9)	859 (86.2)
Ability to transfer	2862 (90.8)	1954 (90.6)	908 (91.2)
Falls	287 (9.1)	203 (9.4)	84 (8.4)
ADL score	8 (5, 8)	6.4 ± 2.1	6.6 ± 2.0
Response mode			
Mail	2592 (82.2)	1742 (80.8)	850 (85.3)
Telephone	561 (17.8)	415 (19.2)	146 (14.7)

Top box score defined as 9-10 on the global rating scale. Data presented as n (%), mean ± standard deviation, or median (25th, 75th percentiles). ESRD: End stage renal disease; BMI: Body mass index; ADL: Activities of daily living; Kt/V: Measure of dialysis adequacy. Unexcused absence was defined as missing an entire HD treatment without rescheduling; shortened treatment was defined as treatments that were at least 15 minutes shorter than prescribed; hospitalization included hospital stays for any reason.

Table 3d: Patient Characteristics stratified by higher or lower Nephrologists' Communication and Caring (NCC) score

	Total (n=3,357)	Top Box (n=1,255, 37%)	Below Top Box (n=2,102, 63%)
Age (years)	62.1 ± 13.9	62.6 ± 13.3	61.8 ± 14.2
Female	1543 (46.0)	616 (49.1)	927 (44.1)
Race			
Black	1292 (38.5)	498 (39.7)	794 (37.8)
White	1909 (56.9)	707 (56.3)	1202 (57.2)
Other	156 (4.7)	50 (4.0)	106 (5.0)
Hispanic Ethnicity	176 (5.2)	59 (4.7)	117 (5.6)
Cause of ESRD			
Diabetes	1349 (40.2)	492 (39.2)	857 (40.8)
Hypertension	958 (28.5)	356 (28.4)	602 (28.6)
Other	1050 (31.3)	407 (32.4)	643 (30.6)
Marital status			
Married	1463 (43.6)	565 (45.0)	898 (42.7)
Divorced/Separated	691 (20.6)	259 (20.6)	432 (20.6)
Widowed	472 (14.1)	182 (14.5)	290 (13.8)
Single	731 (21.8)	249 (19.8)	482 (22.9)
Education Level			
Grade School	269 (8.0)	103 (8.2)	166 (7.9)
High School	2077 (61.9)	790 (63.0)	1287 (61.2)
College/Post Graduate	1011 (30.1)	362 (28.8)	649 (30.9)
English speaker	3314 (98.7)	1242 (99.0)	2072 (98.6)
Insurance			
Medicare/Medicaid	955 (28.5)	357 (28.5)	598 (28.5)
Medicare only	1527 (45.5)	574 (45.7)	953 (45.3)
Medicaid only	152 (4.5)	50 (4.0)	102 (4.9)
Other	723 (21.5)	274 (21.8)	449 (21.4)
Active on transplant waitlist	456 (13.6)	192 (15.3)	264 (12.6)
Vascular access			
Fistula	2190 (65.2)	825 (65.7)	1365 (64.9)
Graft	465 (13.9)	161 (12.8)	304 (14.5)
Catheter	702 (20.9)	269 (21.4)	433 (20.6)
Albumin (g/dL)	3.9 ± 0.4	3.9 ± 0.3	3.9 ± 0.4
Hemoglobin (g/dL)	11.2 ± 1.1	11.2 ± 1.1	11.2 ± 1.1
Kt/V	1.63 ± 0.27	1.65 ± 0.27	1.61 ± 0.27
BMI (kg/m^2)	29.2 ± 7.7	29.0 ± 7.3	29.3 ± 7.8
Unexcused absences	475 (14.2)	163 (13.0)	312 (14.8)
Treatments shortened	1473 (43.9)	489 (39.0)	984 (46.8)
Hospitalization	335 (10.0)	124 (9.9)	211 (10.0)
ESRD vintage (months)	37.6 (18.2, 72.1)	39.2 (18.0, 73.9)	37.3 (18.3, 70.7)

ESRD vintage > 12 months before current facility	709 (21.1)	282 (22.5)	427 (20.3)
Ability to ambulate	2848 (84.8)	1064 (84.8)	1784 (84.9)
Ability to transfer	3037 (90.5)	1147 (91.4)	1890 (89.9)
Falls	310 (9.2)	96 (7.7)	214 (10.2)
ADL score	8 (5, 8)	8 (5, 8)	8 (5, 8)
Response mode			
Mail	2795 (83.3)	1762 (83.8)	1033 (82.3)
Telephone	562 (16.7)	340 (16.2)	222 (17.7)

Data presented as n (%), mean $\pm$ standard deviation, or median (25th, 75th percentiles). ESRD: End stage renal disease; BMI: Body mass index; ADL: Activities of daily living; Kt/V: Measure of dialysis adequacy. Unexcused absence was defined as missing an entire HD treatment without rescheduling; shortened treatment was defined as treatments that were at least 15 minutes shorter than prescribed; hospitalization included hospital stays for any reason.

Table 3e: Patient Characteristics stratified by higher or lower Quality of Dialysis Center Care and Operations (DCO) score

	Total (n=3,238)	Top Box (n=508, 16%)	Below Top Box (n=2,730, 84%)
Age (years)	61.9 ± 13.9	64.4 ± 13.5	61.4 ± 13.9
Female	1480 (45.7)	221 (43.5)	1259 (46.1)
Race			
Black	1237 (38.2)	169 (33.3)	1068 (39.1)
White	1847 (57.0)	322 (63.4)	1525 (55.9)
Other	154 (4.8)	17 (3.4)	137 (5.0)
Hispanic Ethnicity	175 (5.4)	31 (6.1)	144 (5.3)
Cause of ESRD			
Diabetes	1295 (40.0)	199 (39.2)	1096 (40.2)
Hypertension	919 (28.4)	152 (29.9)	767 (28.1)
Other	1024 (31.6)	157 (30.9)	867 (31.8)
Marital status			
Married	1407 (43.5)	242 (47.6)	1165 (42.7)
Divorced/Separated	662 (20.4)	93 (18.3)	569 (20.8)
Widowed	448 (13.8)	80 (15.8)	368 (13.5)
Single	721 (22.3)	93 (18.3)	628 (23.0)
Education Level			
Grade School	265 (8.2)	55 (10.8)	210 (7.7)
High School	2001 (61.8)	330 (65.0)	1671 (61.2)
College/Post Graduate	972 (30.0)	123 (24.2)	849 (31.1)
English speaker	3195 (98.7)	501 (98.6)	2694 (98.7)
Insurance			
Medicare/Medicaid	931 (28.8)	134 (26.4)	797 (29.2)
Medicare only	1465 (45.2)	231 (45.5)	1234 (45.2)
Medicaid only	148 (4.6)	23 (4.5)	125 (4.6)
Other	694 (21.4)	120 (23.6)	574 (21.0)
Active on transplant waitlist	446 (13.8)	49 (9.7)	397 (14.5)
Vascular access			
Fistula	2117 (65.4)	325 (64.0)	1792 (65.6)
Graft	451 (13.9)	86 (16.9)	365 (13.4)
Catheter	670 (20.7)	97 (19.1)	573 (21.0)
Albumin (g/dL)	3.9 ± 0.4	3.9 ± 0.3	3.9 ± 0.4
Hemoglobin (g/dL)	11.2 ± 1.2	11.2 ± 1.0	11.2 ± 1.2
Kt/V	1.62 ± 0.27	1.64 ± 0.27	1.62 ± 0.27
BMI (kg/m^2)	29.2 ± 7.7	28.4 ± 6.8	29.3 ± 7.8
Unexcused absences	464 (14.3)	54 (10.6)	410 (15.0)
Treatments shortened	1425 (44.0)	199 (39.2)	1226 (44.9)
Hospitalization	320 (9.9)	42 (8.3)	278 (10.2)
ESRD vintage (months)	38.1 (18.4, 72.4)	31.0 (15.1, 61.7)	39.5 (19.1, 74.3)

ESRD vintage > 12 months before current facility	689 (21.3)	78 (15.4)	611 (22.4)
Ability to ambulate	2756 (85.1)	427 (84.1)	2329 (85.3)
Ability to transfer	2933 (90.6)	463 (91.1)	2470 (90.5)
Falls	293 (9.1)	47 (9.3)	246 (9.0)
ADL score	8 (5, 8)	8 (5, 8)	8 (5, 8)
Response mode			
Mail	2669 (82.4)	2290 (83.9)	379 (74.6)
Telephone	569 (17.6)	440 (16.1)	129 (25.4)

Data presented as n (%), mean $\pm$ standard deviation, or median (25th, 75th percentiles). ESRD: End stage renal disease; BMI: Body mass index; ADL: Activities of daily living; Kt/V: Measure of dialysis adequacy. Unexcused absence was defined as missing an entire HD treatment without rescheduling; shortened treatment was defined as treatments that were at least 15 minutes shorter than prescribed; hospitalization included hospital stays for any reason.

Table 3f: Patient Characteristics stratified by higher or lower Providing Information to Patients (PIP) score

	Total (n=3,185)	Top Box (n=1,098, 34%)	Below Top Box (n=2,087, 66%)
Age (years)	61.8 ± 13.8	59.1 ± 13.5	63.3 ± 13.8
Female	1454 (45.7)	491 (44.7)	963 (46.1)
Race			
Black	1215 (38.2)	420 (38.3)	795 (38.1)
White	1818 (57.1)	619 (56.4)	1199 (57.5)
Other	152 (4.8)	59 (5.4)	93 (4.5)
Hispanic Ethnicity	173 (5.4)	59 (5.4)	114 (5.5)
Cause of ESRD			
Diabetes	1270 (39.9)	439 (40.0)	831 (39.8)
Hypertension	902 (28.3)	313 (28.5)	589 (28.2)
Other	1013 (31.8)	346 (31.5)	667 (32.0)
Marital status			
Married	1382 (43.4)	481 (43.8)	901 (43.2)
Divorced/Separated	650 (20.4)	214 (19.5)	436 (20.9)
Widowed	443 (13.9)	134 (12.2)	309 (14.8)
Single	710 (22.3)	269 (24.5)	441 (21.1)
Education Level			
Grade School	261 (8.2)	91 (8.3)	170 (8.2)
High School	1965 (61.7)	695 (63.3)	1270 (60.9)
College/Post Graduate	959 (30.1)	312 (28.4)	647 (31.0)
English speaker	3142 (98.7)	1085 (98.8)	2057 (98.6)
Insurance			
Medicare/Medicaid	921 (28.9)	583 (27.9)	338 (30.8)
Medicare only	1436 (45.1)	953 (45.7)	483 (44.0)
Medicaid only	148 (4.7)	95 (4.6)	53 (4.8)
Other	680 (21.4)	456 (21.9)	224 (20.4)
Active on transplant waitlist	439 (13.8)	191 (17.4)	248 (11.9)
Vascular access			
Fistula	2084 (65.4)	712 (64.9)	1372 (65.7)
Graft	444 (13.9)	144 (13.1)	300 (14.4)
Catheter	657 (20.6)	242 (22.0)	415 (19.9)
Albumin (g/dL)	3.9 ± 0.4	3.9 ± 0.4	3.9 ± 0.4
Hemoglobin (g/dL)	11.2 ± 1.2	11.2 ± 1.1	11.2 ± 1.2
Kt/V	1.62 ± 0.27	1.61 ± 0.27	1.63 ± 0.27
BMI (kg/m^2)	29.2 ± 7.7	29.7 ± 7.7	28.9 ± 7.6
Unexcused absences	463 (14.5)	155 (14.1)	308 (14.8)
Treatments shortened	1397 (43.9)	472 (43.0)	925 (44.3)
Hospitalization	315 (9.9)	108 (9.8)	207 (9.9)
ESRD vintage (months)	38.1 (18.4, 72.5)	37.8 (18.1, 71.3)	38.2 (18.5, 73.2)

ESRD vintage > 12 months before current facility	682 (21.4)	225 (20.5)	457 (21.9)
Ability to ambulate	2711 (85.1)	970 (88.3)	1741 (83.4)
Ability to transfer	2888 (90.7)	1029 (93.7)	1859 (89.1)
Falls	290 (9.1)	94 (8.6)	196 (9.4)
ADL score	8 (5, 8)	8 (5, 8)	8 (5, 8)
Response mode			
Mail	2618 (82.2)	1752 (84.0)	866 (78.9)
Telephone	567 (17.8)	335 (16.1)	232 (21.1)

Data presented as n (%), mean $\pm$ standard deviation, or median (25th, 75th percentiles). ESRD: End stage renal disease; BMI: Body mass index; ADL: Activities of daily living; Kt/V: Measure of dialysis adequacy. Unexcused absence was defined as missing an entire HD treatment without rescheduling; shortened treatment was defined as treatments that were at least 15 minutes shorter than prescribed; hospitalization included hospital stays for any reason.

Table 4a: Multivariable association of characteristics with higher nephrologist rating with multiple imputation

	Model 1	Model 2
Age, per 5 years	**1.07 (1.03, 1.11)**	**1.07 (1.03, 1.11)**
Female	1.03 (0.88, 1.21)	0.98 (0.82, 1.17)
Race, black vs white	1.07 (0.89, 1.30)	1.05 (0.86, 1.29)
Race, other vs white	0.71 (0.48, 1.05)	0.71 (0.48, 1.05)
Ethnicity, Hispanic vs non-Hispanic	1.51 (1.00, 2.29)	1.42 (0.94, 2.16)
Insurance, Medicare/Medicaid vs Medicare only	1.06 (0.85, 1.31)	1.07 (0.86, 1.33)
Insurance, Medicaid only vs Medicare only	1.03 (0.68, 1.56)	1.11 (0.73, 1.69)
Insurance, Other vs Medicare only	1.00 (0.81, 1.24)	1.02 (0.82, 1.27)
Marital status, married vs single	0.94 (0.74, 1.19)	0.94 (0.74, 1.19)
Marital status, divorced/separated vs single	0.90 (0.70, 1.15)	0.94 (0.73, 1.20)
Marital status, widowed vs single	1.16 (0.83, 1.62)	1.18 (0.85, 1.65)
Education level, grade school vs college or more	1.17 (0.83, 1.65)	1.11 (0.78, 1.57)
Education level, high school vs college or more	1.03 (0.87, 1.24)	1.02 (0.85, 1.22)
English speaker	0.81 (0.38, 1.71)	0.87 (0.41, 1.85)
Hospitalization		0.90 (0.69, 1.19)
Active on transplant waitlist		1.19 (0.93, 1.52)
BMI, per 2 kg/m^2		1.02 (1.00, 1.04)
Cause ESRD, diabetes vs. other		0.90 (0.74, 1.10)
Cause ESRD, hypertension vs. other		0.95 (0.77, 1.19)
Vascular access, catheter vs. fistula		0.95 (0.75, 1.21)
Vascular access, graft vs. fistula		1.05 (0.85, 1.30)
Hemoglobin, per 0.5 g/dL		**0.95 (0.92, 0.98)**
Albumin, per 0.2 g/dL		1.00 (0.96, 1.05)
Kt/V, per 0.2		1.02 (0.95, 1.09)
ESRD vintage, per 12 months		1.02 (0.99, 1.05)
ESRD vintage > 12 months before current facility		0.88 (0.70, 1.11)
Unexcused absences		0.83 (0.66, 1.04)
Treatments shortened		**0.70 (0.59, 0.83)**
Telephone administration vs mail		**1.56 (1.24, 1.98)**

Data shown as odds ratio (OR) (95% CI). Odds ratio above 1.00 is associated with top box response. Associations with $p<0.01$ are in bold. ESRD: End stage renal disease; BMI: Body mass index; ADL: Activities of daily living; Kt/V: Measure of dialysis adequacy. Unexcused absence was defined as missing an entire HD treatment without rescheduling; shortened treatment was defined as treatments that were at least 15 minutes shorter than prescribed; hospitalization included hospital stays for any reason.

Table 4b: Multivariable association of characteristics with higher dialysis staff rating with multiple imputation

	Model 1	Model 2
Age, per 5 years	**1.10 (1.06, 1.15)**	**1.09 (1.04, 1.13)**
Female	0.87 (0.72, 1.04)	0.82 (0.67, 1.00)
Race, black vs white	0.88 (0.71, 1.08)	0.87 (0.70, 1.09)
Race, other vs white	0.74 (0.47, 1.14)	0.73 (0.47, 1.14)
Ethnicity, Hispanic vs non-Hispanic	1.21 (0.77, 1.90)	1.11 (0.70, 1.74)
Insurance, Medicare/Medicaid vs Medicare only	0.93 (0.73, 1.17)	0.92 (0.72, 1.17)
Insurance, Medicaid only vs Medicare only	1.51 (0.94, 2.44)	1.56 (0.96, 2.53)
Insurance, Other vs Medicare only	1.01 (0.79, 1.29)	0.96 (0.74, 1.23)
Marital status, married vs single	1.07 (0.83, 1.39)	1.07 (0.82, 1.39)
Marital status, divorced/separated vs single	0.94 (0.72, 1.23)	0.97 (0.74, 1.27)
Marital status, widowed vs single	1.41 (0.97, 2.04)	1.42 (0.97, 2.06)
Education level, grade school vs college or more	1.22 (0.83, 1.79)	1.10 (0.75, 1.63)
Education level, high school vs college or more	1.16 (0.95, 1.42)	1.13 (0.92, 1.39)
English speaker	0.96 (0.46, 2.02)	1.02 (0.49, 2.14)
Hospitalization		1.06 (0.76, 1.46)
Active on transplant waitlist		1.09 (0.83, 1.44)
BMI, per 2 kg/m^2		1.01 (0.98, 1.03)
Cause ESRD, diabetes vs. other		1.02 (0.81, 1.28)
Cause ESRD, hypertension vs. other		1.06 (0.83, 1.35)
Vascular access, catheter vs. fistula		0.90 (0.68, 1.18)
Vascular access, graft vs. fistula		1.13 (0.89, 1.43)
Hemoglobin, per 0.5 g/dL		0.98 (0.94, 1.02)
Albumin, per 0.2 g/dL		0.96 (0.91, 1.01)
Kt/V, per 0.2		1.05 (0.97, 1.13)
ESRD vintage, per 12 months		0.98 (0.94, 1.01)
ESRD vintage > 12 months before current facility		0.92 (0.72, 1.19)
Unexcused absences		0.87 (0.68, 1.12)
Treatments shortened		**0.76 (0.63, 0.92)**
Telephone administration vs mail		**1.72 (1.32, 2.23)**

Data shown as odds ratio (OR) (95% CI). Odds ratio above 1.00 is associated with top box response. Associations with $p<0.01$ are in bold. ESRD: End stage renal disease; BMI: Body mass index; ADL: Activities of daily living; Kt/V: Measure of dialysis adequacy. Unexcused absence was defined as missing an entire HD treatment without rescheduling; shortened treatment was defined as treatments that were at least 15 minutes shorter than prescribed; hospitalization included hospital stays for any reason.

Table 4c: Multivariable association of characteristics with higher dialysis facility rating with multiple imputation

	Model 1	Model 2
Age, per 5 years	**1.12 (1.08, 1.16)**	**1.10 (1.05, 1.15)**
Female	0.91 (0.76, 1.09)	0.82 (0.67, 1.00)
Race, black vs white	0.85 (0.69, 1.05)	0.84 (0.67, 1.05)
Race, other vs white	0.81 (0.52, 1.27)	0.79 (0.50, 1.25)
Ethnicity, Hispanic vs non-Hispanic	1.47 (0.90, 2.40)	1.31 (0.80, 2.15)
Insurance, Medicare/Medicaid vs Medicare only	1.09 (0.86, 1.38)	1.05 (0.82, 1.34)
Insurance, Medicaid only vs Medicare only	1.06 (0.68, 1.65)	1.06 (0.68, 1.67)
Insurance, Other vs Medicare only	1.07 (0.83, 1.37)	1.02 (0.79, 1.32)
Marital status, married vs single	1.02 (0.78, 1.33)	1.02 (0.78, 1.33)
Marital status, divorced/separated vs single	0.91 (0.70, 1.19)	0.95 (0.72, 1.25)
Marital status, widowed vs single	1.39 (0.95, 2.04)	1.39 (0.94, 2.05)
Education level, grade school vs college or more	1.26 (0.85, 1.88)	1.13 (0.75, 1.70)
Education level, high school vs college or more	1.13 (0.92, 1.38)	1.10 (0.89, 1.35)
English speaker	0.90 (0.38, 2.10)	0.96 (0.42, 2.20)
Hospitalization		0.92 (0.67, 1.27)
Active on transplant waitlist		0.95 (0.72, 1.25)
BMI, per 2 kg/m^2		1.01 (0.99, 1.04)
Cause ESRD, diabetes vs. other		0.99 (0.79, 1.25)
Cause ESRD, hypertension vs. other		1.00 (0.78, 1.28)
Vascular access, catheter vs. fistula		0.93 (0.71, 1.23)
Vascular access, graft vs. fistula		1.15 (0.90, 1.46)
Hemoglobin, per 0.5 g/dL		0.96 (0.93, 1.00)
Albumin, per 0.2 g/dL		**0.94 (0.90, 1.00)**
Kt/V, per 0.2		**1.09 (1.01, 1.17)**
ESRD vintage, per 12 months		0.97 (0.94, 1.01)
ESRD vintage > 12 months before current facility		1.08 (0.84, 1.40)
Unexcused absences		0.89 (0.69, 1.14)
Treatments shortened		**0.74 (0.62, 0.90)**
Telephone administration vs mail		**2.00 (1.51, 2.64)**

Data shown as odds ratio (OR) (95% CI). Odds ratio above 1.00 is associated with top box response. Associations with p<0.01 are in bold. ESRD: End stage renal disease; BMI: Body mass index; ADL: Activities of daily living; Kt/V: Measure of dialysis adequacy. Unexcused absence was defined as missing an entire HD treatment without rescheduling; shortened treatment was defined as treatments that were at least 15 minutes shorter than prescribed; hospitalization included hospital stays for any reason.

Table 4d: Multivariable association of characteristics with higher Nephrologists' Communication and Caring (NCC) score with multiple imputation

	Model 1	Model 2
Age, per 5 years	1.01 (0.98, 1.04)	1.01 (0.98, 1.04)
Female	**1.18 (1.03, 1.36)**	1.13 (0.97, 1.31)
Race, black vs white	1.09 (0.93, 1.28)	1.13 (0.96, 1.33)
Race, other vs white	0.82 (0.58, 1.16)	0.79 (0.56, 1.14)
Ethnicity, Hispanic vs non-Hispanic	1.07 (0.76, 1.51)	1.01 (0.71, 1.44)
Insurance, Medicare/Medicaid vs Medicare only	1.06 (0.89, 1.27)	1.10 (0.91, 1.32)
Insurance, Medicaid only vs Medicare only	0.95 (0.66, 1.36)	1.00 (0.69, 1.44)
Insurance, Other vs Medicare only	1.04 (0.87, 1.24)	1.05 (0.88, 1.26)
Marital status, married vs single	1.18 (0.96, 1.45)	1.18 (0.96, 1.45)
Marital status, divorced/separated vs single	1.08 (0.87, 1.34)	1.11 (0.89, 1.37)
Marital status, widowed vs single	1.06 (0.81, 1.38)	1.05 (0.80, 1.38)
Education level, grade school vs college or more	1.12 (0.85, 1.47)	1.12 (0.85, 1.49)
Education level, high school vs college or more	1.06 (0.91, 1.23)	1.07 (0.92, 1.25)
English speaker	1.35 (0.76, 2.38)	1.39 (0.78, 2.49)
Hospitalization		1.06 (0.84, 1.34)
Active on transplant waitlist		**1.29 (1.05, 1.58)**
BMI, per 2 kg/m^2		1.00 (0.98, 1.02)
Cause ESRD, diabetes vs. other		0.92 (0.77, 1.09)
Cause ESRD, hypertension vs. other		0.95 (0.79, 1.14)
Vascular access, catheter vs. fistula		0.93 (0.76, 1.14)
Vascular access, graft vs. fistula		0.90 (0.76, 1.08)
Hemoglobin, per 0.5 g/dL		0.99 (0.96, 1.02)
Albumin, per 0.2 g/dL		1.01 (0.97, 1.05)
Kt/V, per 0.2		**1.08 (1.02, 1.14)**
ESRD vintage, per 12 months		1.00 (0.97, 1.03)
ESRD vintage > 12 months before current facility		1.10 (0.91, 1.33)
Unexcused absences		0.90 (0.73, 1.10)
Treatments shortened		**0.77 (0.67, 0.89)**
Telephone administration vs mail		1.16 (0.97, 1.40)

Data shown as odds ratio (OR) (95% CI). Odds ratio above 1.00 is associated with top box response. Associations with p<0.01 are in bold. ESRD: End stage renal disease; BMI: Body mass index; ADL: Activities of daily living; Kt/V: Measure of dialysis adequacy. Unexcused absence was defined as missing an entire HD treatment without rescheduling; shortened treatment was defined as treatments that were at least 15 minutes shorter than prescribed; hospitalization included hospital stays for any reason.

Table 4e: Multivariable association of characteristics with higher Quality of Dialysis Center Care and Operations (DCO) score with multiple imputation

	Model 1	Model 2
Age, per 5 years	**1.08 (1.03, 1.12)**	1.04 (0.99, 1.09)
Female	0.86 (0.71, 1.04)	0.86 (0.70, 1.06)
Race, black vs white	0.88 (0.71, 1.09)	0.90 (0.71, 1.13)
Race, other vs white	0.66 (0.40, 1.11)	0.70 (0.42, 1.18)
Ethnicity, Hispanic vs non-Hispanic	1.33 (0.86, 2.05)	1.10 (0.70, 1.73)
Insurance, Medicare/Medicaid vs Medicare only	1.13 (0.88, 1.45)	1.10 (0.85, 1.42)
Insurance, Medicaid only vs Medicare only	1.17 (0.71, 1.91)	1.13 (0.68, 1.88)
Insurance, Other vs Medicare only	1.20 (0.95, 1.52)	1.12 (0.88, 1.43)
Marital status, married vs single	1.12 (0.84, 1.49)	1.22 (0.91, 1.64)
Marital status, divorced/separated vs single	1.02 (0.75, 1.38)	1.12 (0.82, 1.52)
Marital status, widowed vs single	1.08 (0.75, 1.56)	1.13 (0.78, 1.63)
Education level, grade school vs college or more	**1.81 (1.26, 2.59)**	**1.64 (1.13, 2.38)**
Education level, high school vs college or more	**1.50 (1.20, 1.86)**	**1.46 (1.17, 1.82)**
English speaker	1.34 (0.60, 2.97)	1.50 (0.65, 3.48)
Hospitalization		0.78 (0.56, 1.09)
Active on transplant waitlist		0.74 (0.54, 1.00)
BMI, per 2 kg/m^2		0.97 (0.95, 1.00)
Cause ESRD, diabetes vs. other		0.97 (0.77, 1.22)
Cause ESRD, hypertension vs. other		1.01 (0.78, 1.29)
Vascular access, catheter vs. fistula		**1.43 (1.10, 1.85)**
Vascular access, graft vs. fistula		0.96 (0.75, 1.22)
Hemoglobin, per 0.5 g/dL		0.99 (0.94, 1.03)
Albumin, per 0.2 g/dL		1.02 (0.97, 1.08)
Kt/V, per 0.2		1.05 (0.97, 1.13)
ESRD vintage, per 12 months		**0.96 (0.92, 0.99)**
ESRD vintage > 12 months before current facility		0.76 (0.57, 1.01)
Unexcused absences		**0.67 (0.50, 0.91)**
Treatments shortened		0.86 (0.71, 1.05)
Telephone administration vs mail		**1.93 (1.53, 2.43)**

Data shown as odds ratio (OR) (95% CI). Odds ratio above 1.00 is associated with top box response. Associations with p<0.01 are in bold. ESRD: End stage renal disease; BMI: Body mass index; ADL: Activities of daily living; Kt/V: Measure of dialysis adequacy. Unexcused absence was defined as missing an entire HD treatment without rescheduling; shortened treatment was defined as treatments that were at least 15 minutes shorter than prescribed; hospitalization included hospital stays for any reason.

Table 4f: Multivariable association of characteristics with higher Providing Information to Patients (PIP) score with multiple imputation

	Model 1	Model 2
Age, per 5 years	**0.86 (0.84, 0.89)**	**0.87 (0.84, 0.90)**
Female	0.96 (0.83, 1.11)	1.00 (0.85, 1.17)
Race, black vs white	0.90 (0.76, 1.06)	0.85 (0.72, 1.02)
Race, other vs white	1.20 (0.84, 1.70)	1.23 (0.86, 1.76)
Ethnicity, Hispanic vs non-Hispanic	0.85 (0.60, 1.22)	0.79 (0.55, 1.14)
Insurance, Medicare/Medicaid vs Medicare only	0.95 (0.79, 1.15)	0.97 (0.80, 1.18)
Insurance, Medicaid only vs Medicare only	0.80 (0.55, 1.17)	0.85 (0.58, 1.24)
Insurance, Other vs Medicare only	0.92 (0.76, 1.12)	0.89 (0.73, 1.08)
Marital status, married vs single	**1.30 (1.05, 1.60)**	**1.29 (1.04, 1.60)**
Marital status, divorced/separated vs single	1.03 (0.82, 1.29)	1.04 (0.83, 1.31)
Marital status, widowed vs single	1.33 (1.00, 1.77)	1.33 (1.00, 1.78)
Education level, grade school vs college or more	1.14 (0.85, 1.53)	1.09 (0.81, 1.47)
Education level, high school vs college or more	1.08 (0.92, 1.26)	1.07 (0.91, 1.25)
English speaker	1.20 (0.71, 2.02)	1.25 (0.74, 2.11)
Hospitalization		1.02 (0.80, 1.31)
Active on transplant waitlist		**1.38 (1.10, 1.72)**
BMI, per 2 kg/m^2		1.01 (0.99, 1.03)
Cause ESRD, diabetes vs. other		1.09 (0.91, 1.31)
Cause ESRD, hypertension vs. other		1.13 (0.93, 1.38)
Vascular access, catheter vs. fistula		1.03 (0.83, 1.28)
Vascular access, graft vs. fistula		1.16 (0.96, 1.39)
Hemoglobin, per 0.5 g/dL		1.00 (0.96, 1.03)
Albumin, per 0.2 g/dL		1.04 (1.00, 1.09)
Kt/V, per 0.2		0.96 (0.90, 1.01)
ESRD vintage, per 12 months		0.99 (0.96, 1.02)
ESRD vintage > 12 months before current facility		0.87 (0.71, 1.07)
Unexcused absences		0.84 (0.68, 1.04)
Treatments shortened		**0.84 (0.72, 0.98)**
Telephone administration vs mail		**1.36 (1.13, 1.65)**

Data shown as odds ratio (OR) (95% CI). Odds ratio above 1.00 is associated with top box response. Associations with p<0.01 are in bold. ESRD: End stage renal disease; BMI: Body mass index; ADL: Activities of daily living; Kt/V: Measure of dialysis adequacy. Unexcused absence was defined as missing an entire HD treatment without rescheduling; shortened treatment was defined as treatments that were at least 15 minutes shorter than prescribed; hospitalization included hospital stays for any reason.

Table 5a: Multivariable association of characteristics with higher nephrologist rating using older top box definition*

	Model 1	Model 2
ICC	0.06	0.06
Age, per 5 years	**1.07 (1.03, 1.10)**	**1.06 (1.02, 1.10)**
Female	**1.26 (1.08, 1.46)**	**1.21 (1.02, 1.43)**
Race, black vs white	1.18 (0.99, 1.41)	**1.21 (1.01, 1.46)**
Race, other vs white	0.80 (0.54, 1.16)	0.79 (0.54, 1.16)
Ethnicity, Hispanic vs non-Hispanic	1.05 (0.71, 1.55)	1.03 (0.69, 1.54)
Insurance, Medicare/Medicaid vs Medicare only	1.03 (0.85, 1.26)	1.09 (0.89, 1.33)
Insurance, Medicaid only vs Medicare only	0.91 (0.62, 1.34)	0.99 (0.67, 1.46)
Insurance, Other vs Medicare only	1.06 (0.87, 1.28)	1.07 (0.88, 1.31)
Marital status, married vs single	1.10 (0.88, 1.37)	1.11 (0.89, 1.38)
Marital status, divorced/separated vs single	1.00 (0.80, 1.26)	1.06 (0.84, 1.33)
Marital status, widowed vs single	1.14 (0.85, 1.54)	1.17 (0.87, 1.58)
Education level, grade school vs college or more	1.35 (0.99, 1.85)	1.35 (0.98, 1.86)
Education level, high school vs college or more	**1.19 (1.01, 1.40)**	**1.19 (1.01, 1.41)**
English speaker	**0.43 (0.18, 0.98)**	0.48 (0.21, 1.11)
Hospitalization		0.93 (0.73, 1.20)
Active on transplant waitlist		1.25 (0.99, 1.57)
BMI, per 2 kg/m^2		1.01 (0.99, 1.03)
Cause ESRD, diabetes vs. other		0.85 (0.71, 1.03)
Cause ESRD, hypertension vs. other		1.02 (0.83, 1.25)
Vascular access, catheter vs. fistula		0.88 (0.70, 1.10)
Vascular access, graft vs. fistula		0.97 (0.80, 1.18)
Hemoglobin, per 0.5 g/dL		0.98 (0.95, 1.01)
Albumin, per 0.2 g/dL		0.99 (0.95, 1.04)
Kt/V, per 0.2		1.06 (0.99, 1.13)
ESRD vintage, per 12 months		0.99 (0.97, 1.02)
ESRD vintage > 12 months before current facility		0.95 (0.77, 1.18)
Unexcused absences		**0.77 (0.62, 0.96)**
Treatments shortened		**0.71 (0.61, 0.83)**
Telephone administration vs mail		1.20 (0.97, 1.47)

*Prior to 2014 top box referred to 8-10 rating instead of 9-10. Data shown as odds ratio (OR) (95% CI). Odds ratio above 1.00 is associated with top box response. Associations with p<0.01 are in bold. ESRD: End stage renal disease; BMI: Body mass index; ADL: Activities of daily living; Kt/V: Measure of dialysis adequacy. Unexcused absence was defined as missing an entire HD treatment without rescheduling; shortened treatment was defined as treatments that were at least 15 minutes shorter than prescribed; hospitalization included hospital stays for any reason.

Table 5b: Multivariable association of characteristics with higher dialysis staff rating using older top box definition*

	Model 1	Model 2
ICC	0.08	0.08
Age, per 5 years	**1.07 (1.04, 1.11)**	**1.06 (1.03, 1.10)**
Female	0.99 (0.84, 1.16)	0.95 (0.80, 1.13)
Race, black vs white	0.95 (0.79, 1.14)	0.92 (0.76, 1.12)
Race, other vs white	0.92 (0.62, 1.37)	0.92 (0.61, 1.37)
Ethnicity, Hispanic vs non-Hispanic	0.87 (0.58, 1.30)	0.82 (0.54, 1.23)
Insurance, Medicare/Medicaid vs Medicare only	0.96 (0.78, 1.19)	0.96 (0.78, 1.18)
Insurance, Medicaid only vs Medicare only	1.50 (0.99, 2.27)	**1.54 (1.02, 2.34)**
Insurance, Other vs Medicare only	1.02 (0.83, 1.25)	1.00 (0.81, 1.23)
Marital status, married vs single	1.07 (0.85, 1.34)	1.06 (0.84, 1.33)
Marital status, divorced/separated vs single	1.00 (0.79, 1.27)	1.02 (0.80, 1.30)
Marital status, widowed vs single	1.21 (0.88, 1.65)	1.21 (0.88, 1.66)
Education level, grade school vs college or more	**1.72 (1.23, 2.41)**	**1.61 (1.15, 2.27)**
Education level, high school vs college or more	**1.45 (1.22, 1.72)**	**1.42 (1.19, 1.70)**
English speaker	1.02 (0.48, 2.15)	1.14 (0.54, 2.41)
Hospitalization		1.03 (0.79, 1.35)
Active on transplant waitlist		1.03 (0.81, 1.31)
BMI, per 2 kg/m^2		1.01 (0.99, 1.04)
Cause ESRD, diabetes vs. other		1.05 (0.86, 1.27)
Cause ESRD, hypertension vs. other		1.05 (0.85, 1.29)
Vascular access, catheter vs. fistula		0.84 (0.66, 1.06)
Vascular access, graft vs. fistula		**1.24 (1.01, 1.53)**
Hemoglobin, per 0.5 g/dL		1.00 (0.97, 1.04)
Albumin, per 0.2 g/dL		1.01 (0.96, 1.06)
Kt/V, per 0.2		1.05 (0.98, 1.12)
ESRD vintage, per 12 months		0.98 (0.95, 1.01)
ESRD vintage > 12 months before current facility		0.90 (0.72, 1.13)
Unexcused absences		0.96 (0.76, 1.20)
Treatments shortened		**0.81 (0.69, 0.96)**
Telephone administration vs mail		**1.33 (1.07, 1.65)**

*Prior to 2014 top box referred to 8-10 rating instead of 9-10. Data shown as odds ratio (OR) (95% CI). Odds ratio above 1.00 is associated with top box response. Associations with $p<0.01$ are in bold. ESRD: End stage renal disease; BMI: Body mass index; ADL: Activities of daily living; Kt/V: Measure of dialysis adequacy. Unexcused absence was defined as missing an entire HD treatment without rescheduling; shortened treatment was defined as treatments that were at least 15 minutes shorter than prescribed; hospitalization included hospital stays for any reason.

Table 5c: Multivariable association of characteristics with higher dialysis facility rating using older top box definition*

	Model 1	Model 2
ICC	0.09	0.08
Age, per 5 years	**1.08 (1.04, 1.12)**	**1.07 (1.03, 1.11)**
Female	0.97 (0.82, 1.14)	0.93 (0.78, 1.11)
Race, black vs white	1.04 (0.86, 1.27)	1.05 (0.86, 1.29)
Race, other vs white	1.03 (0.68, 1.57)	1.07 (0.70, 1.63)
Ethnicity, Hispanic vs non-Hispanic	0.95 (0.63, 1.45)	0.88 (0.58, 1.35)
Insurance, Medicare/Medicaid vs Medicare only	0.98 (0.79, 1.21)	0.97 (0.78, 1.21)
Insurance, Medicaid only vs Medicare only	1.07 (0.71, 1.62)	1.09 (0.72, 1.66)
Insurance, Other vs Medicare only	0.99 (0.80, 1.23)	0.94 (0.76, 1.16)
Marital status, married vs single	1.15 (0.91, 1.44)	1.17 (0.92, 1.48)
Marital status, divorced/separated vs single	1.10 (0.87, 1.41)	1.15 (0.90, 1.47)
Marital status, widowed vs single	1.26 (0.92, 1.74)	1.30 (0.94, 1.80)
Education level, grade school vs college or more	**1.99 (1.40, 2.83)**	**1.88 (1.31, 2.70)**
Education level, high school vs college or more	**1.45 (1.21, 1.73)**	**1.43 (1.20, 1.71)**
English speaker	**0.31 (0.11, 0.86)**	**0.35 (0.13, 0.98)**
Hospitalization		0.90 (0.69, 1.19)
Active on transplant waitlist		1.00 (0.79, 1.27)
BMI, per 2 kg/m^2		1.02 (0.99, 1.04)
Cause ESRD, diabetes vs. other		0.99 (0.81, 1.21)
Cause ESRD, hypertension vs. other		0.97 (0.78, 1.20)
Vascular access, catheter vs. fistula		1.11 (0.87, 1.43)
Vascular access, graft vs. fistula		1.20 (0.97, 1.49)
Hemoglobin, per 0.5 g/dL		1.00 (0.97, 1.04)
Albumin, per 0.2 g/dL		1.00 (0.95, 1.05)
Kt/V, per 0.2		1.03 (0.96, 1.10)
ESRD vintage, per 12 months		0.97 (0.94, 1.00)
ESRD vintage > 12 months before current facility		0.91 (0.73, 1.14)
Unexcused absences		0.88 (0.70, 1.11)
Treatments shortened		**0.81 (0.69, 0.96)**
Telephone administration vs mail		**1.45 (1.16, 1.81)**

*Prior to 2014 top box referred to 8-10 rating instead of 9-10. Data shown as odds ratio (OR) (95% CI). Odds ratio above 1.00 is associated with top box response. Associations with p<0.01 are in bold. ESRD: End stage renal disease; BMI: Body mass index; ADL: Activities of daily living; Kt/V: Measure of dialysis adequacy. Unexcused absence was defined as missing an entire HD treatment without rescheduling; shortened treatment was defined as treatments that were at least 15 minutes shorter than prescribed; hospitalization included hospital stays for any reason.

Table 6: Multivariable association of characteristics with higher scores after excluding patients who responded by phone

	Nephrologist Rating (N=3294)	Staff Rating (N=3145)	Dialysis Facility Rating (N=3153)	NCC Score (N=3357)	DCO Score (N=3238)	PIP Score (N=3185)
ICC	0.06	0.09	0.08	0.03	0.06	0.04
Age, per 5 years	**1.08 (1.03, 1.13)**	**1.10 (1.04, 1.15)**	**1.11 (1.05, 1.17)**	1.03 (0.99, 1.07)	1.05 (0.99, 1.11)	**0.86 (0.82, 0.89)**
Female	1.07 (0.87, 1.32)	0.84 (0.66, 1.06)	0.81 (0.64, 1.02)	**1.24 (1.04, 1.48)**	0.88 (0.68, 1.14)	1.06 (0.87, 1.28)
Race, black vs white	1.04 (0.83, 1.30)	0.91 (0.70, 1.17)	0.87 (0.68, 1.13)	1.10 (0.91, 1.34)	0.79 (0.59, 1.05)	**0.78 (0.64, 0.97)**
Race, other vs white	0.73 (0.46, 1.16)	0.95 (0.55, 1.64)	0.96 (0.55, 1.67)	0.80 (0.52, 1.24)	**0.45 (0.21, 0.94)**	1.39 (0.89, 2.17)
Ethnicity, Hispanic vs non-Hispanic	1.32 (0.77, 2.24)	0.80 (0.45, 1.42)	1.03 (0.57, 1.87)	0.88 (0.55, 1.41)	1.46 (0.79, 2.71)	**0.53 (0.31, 0.89)**
Insurance, Medicare/Medicaid vs Medicare only	0.96 (0.74, 1.24)	0.90 (0.68, 1.19)	1.02 (0.77, 1.35)	1.04 (0.84, 1.29)	1.18 (0.85, 1.64)	0.83 (0.65, 1.04)
Insurance, Medicaid only vs Medicare only	0.95 (0.59, 1.54)	1.49 (0.84, 2.62)	1.04 (0.61, 1.75)	0.83 (0.53, 1.29)	1.18 (0.62, 2.25)	0.66 (0.42, 1.06)
Insurance, Other vs Medicare only	0.90 (0.70, 1.15)	0.93 (0.69, 1.24)	0.92 (0.69, 1.22)	0.99 (0.80, 1.22)	1.05 (0.79, 1.40)	0.86 (0.68, 1.08)
Marital status, married vs single	0.93 (0.70, 1.23)	1.09 (0.80, 1.48)	1.06 (0.78, 1.44)	1.12 (0.87, 1.43)	1.22 (0.85, 1.76)	1.12 (0.87, 1.45)
Marital status, divorced/separated vs single	0.87 (0.65, 1.16)	0.87 (0.63, 1.18)	0.87 (0.63, 1.18)	1.06 (0.82, 1.37)	1.03 (0.70, 1.53)	0.83 (0.63, 1.09)
Marital status, widowed vs single	1.04 (0.71, 1.52)	1.34 (0.86, 2.07)	1.41 (0.90, 2.20)	0.89 (0.64, 1.22)	1.05 (0.66, 1.68)	1.22 (0.87, 1.73)
Education level, grade school vs college or more	1.32 (0.84, 2.08)	1.45 (0.85, 2.48)	1.32 (0.79, 2.23)	1.21 (0.84, 1.74)	**1.79 (1.10, 2.91)**	1.21 (0.82, 1.80)
Education level, high school vs college or more	1.05 (0.85, 1.29)	1.10 (0.87, 1.39)	1.09 (0.86, 1.38)	1.09 (0.92, 1.30)	**1.41 (1.09, 1.84)**	1.05 (0.87, 1.28)
Hospitalization	0.89 (0.64, 1.23)	1.06 (0.72, 1.55)	1.00 (0.68, 1.47)	0.95 (0.71, 1.27)	0.67 (0.42, 1.06)	0.97 (0.71, 1.33)
Active on transplant waitlist	1.25 (0.94, 1.66)	1.03 (0.76, 1.40)	0.92 (0.68, 1.24)	1.21 (0.95, 1.53)	**0.67 (0.45, 0.99)**	**1.39 (1.08, 1.77)**
BMI, per 2 kg/m2	1.02 (0.99,	1.02 (0.99,	1.02 (0.99,	1.00 (0.98,	0.98 (0.94,	1.01 (0.99,

Cause ESRD, diabetes vs. other	0.85 (0.67, 1.08)	1.00 (0.77, 1.31)	0.97 (0.74, 1.27)	0.91 (0.75, 1.11)	0.96 (0.72, 1.28)	1.09 (0.88, 1.35)
Cause ESRD, hypertension vs. other	0.88 (0.68, 1.13)	0.98 (0.74, 1.30)	0.88 (0.66, 1.16)	0.93 (0.75, 1.15)	1.12 (0.83, 1.51)	1.23 (0.98, 1.55)
Vascular access, catheter vs. fistula	0.97 (0.73, 1.29)	0.82 (0.59, 1.13)	0.87 (0.63, 1.21)	0.92 (0.72, 1.17)	1.36 (0.98, 1.88)	1.03 (0.79, 1.35)
Vascular access, graft vs. fistula	1.02 (0.80, 1.30)	1.13 (0.85, 1.50)	1.27 (0.95, 1.68)	0.96 (0.78, 1.18)	1.00 (0.74, 1.35)	1.24 (1.00, 1.55)
Hemoglobin, per 0.5 g/dL	**0.95 (0.91, 0.99)**	1.00 (0.95, 1.04)	0.99 (0.94, 1.04)	1.01 (0.97, 1.04)	0.98 (0.93, 1.04)	1.01 (0.97, 1.05)
Albumin, per 0.2 g/dL	1.00 (0.94, 1.05)	0.96 (0.90, 1.02)	0.95 (0.89, 1.01)	1.02 (0.97, 1.07)	1.05 (0.98, 1.13)	1.03 (0.98, 1.08)
Kt/V, per 0.2	1.02 (0.94, 1.10)	1.04 (0.95, 1.13)	1.07 (0.98, 1.17)	**1.09 (1.02, 1.16)**	1.07 (0.97, 1.17)	0.94 (0.87, 1.01)
ESRD vintage, per 12 months	1.01 (0.98, 1.05)	0.96 (0.92, 1.00)	**0.96 (0.92, 1.00)**	1.00 (0.97, 1.03)	**0.94 (0.90, 0.99)**	0.99 (0.95, 1.02)
ESRD vintage > 12 months before current facility	0.87 (0.67, 1.13)	0.91 (0.68, 1.21)	1.00 (0.75, 1.34)	1.14 (0.91, 1.43)	**0.68 (0.47, 0.99)**	0.84 (0.66, 1.08)
Unexcused absences	0.80 (0.62, 1.05)	0.95 (0.70, 1.28)	0.87 (0.65, 1.17)	0.99 (0.77, 1.27)	0.74 (0.50, 1.08)	0.90 (0.69, 1.16)
Treatments shortened	**0.70 (0.58, 0.85)**	**0.75 (0.60, 0.94)**	**0.72 (0.58, 0.90)**	**0.71 (0.60, 0.84)**	0.99 (0.78, 1.27)	0.85 (0.71, 1.02)

Data shown as odds ratio (OR) (95% CI) adjusted for all other variables in the table. Odds ratio above 1.00 is associated with top box response. Associations with p<0.01 are in bold. ESRD: End stage renal disease; BMI: Body mass index; ADL: Activities of daily living; Kt/V: Measure of dialysis adequacy. Unexcused absence was defined as missing an entire HD treatment without rescheduling; shortened treatment was defined as treatments that were at least 15 minutes shorter than prescribed; hospitalization included hospital stays for any reason.
